# MCC950/CRID3 potently targets the NACHT domain of wild-type NLRP3 but not disease-associated mutants for inflammasome inhibition

**DOI:** 10.1371/journal.pbio.3000354

**Published:** 2019-09-16

**Authors:** Lieselotte Vande Walle, Irma B. Stowe, Pavel Šácha, Bettina L. Lee, Dieter Demon, Amelie Fossoul, Filip Van Hauwermeiren, Pedro H. V. Saavedra, Petr Šimon, Vladimír Šubrt, Libor Kostka, Craig E. Stivala, Victoria C. Pham, Steven T. Staben, Sayumi Yamazoe, Jan Konvalinka, Nobuhiko Kayagaki, Mohamed Lamkanfi

**Affiliations:** 1 Inflammation Research Center, VIB, Ghent, Belgium; 2 Department of Internal Medicine and Pediatrics, Ghent University, Ghent, Belgium; 3 Discovery Sciences, Janssen Research & Development, Pharmaceutical Companies of Johnson & Johnson, Beerse, Belgium; 4 Department of Physiological Chemistry, Genentech, South San Francisco, California, United States of America; 5 Institute of Organic Chemistry and Biochemistry of The Czech Academy of Sciences, Prague, Czech Republic; 6 Janssen Immunosciences, World Without Disease Accelerator, Pharmaceutical Companies of Johnson & Johnson, Beerse, Belgium; 7 Institute of Macromolecular Chemistry, Academy of Science of the Czech Republic, Prague, Czech Republic; 8 Department of Discovery Chemistry, Genentech, South San Francisco, California, United States of America; New York University School of Medicine, UNITED STATES

## Abstract

The nucleotide-binding-domain (NBD)–and leucine-rich repeat (LRR)–containing (NLR) family, pyrin-domain–containing 3 (NLRP3) inflammasome drives pathological inflammation in a suite of autoimmune, metabolic, malignant, and neurodegenerative diseases. Additionally, *NLRP3* gain-of-function point mutations cause systemic periodic fever syndromes that are collectively known as cryopyrin-associated periodic syndrome (CAPS). There is significant interest in the discovery and development of diarylsulfonylurea Cytokine Release Inhibitory Drugs (CRIDs) such as MCC950/CRID3, a potent and selective inhibitor of the NLRP3 inflammasome pathway, for the treatment of CAPS and other diseases. However, drug discovery efforts have been constrained by the lack of insight into the molecular target and mechanism by which these CRIDs inhibit the NLRP3 inflammasome pathway. Here, we show that the NAIP, CIITA, HET-E, and TP1 (NACHT) domain of NLRP3 is the molecular target of diarylsulfonylurea inhibitors. Interestingly, we find photoaffinity labeling (PAL) of the NACHT domain requires an intact (d)ATP-binding pocket and is substantially reduced for most CAPS-associated NLRP3 mutants. In concordance with this finding, MCC950/CRID3 failed to inhibit NLRP3-driven inflammatory pathology in two mouse models of CAPS. Moreover, it abolished circulating levels of interleukin (IL)-1β and IL-18 in lipopolysaccharide (LPS)-challenged wild-type mice but not in *Nlrp3*^L351P^ knock-in mice and ex vivo-stimulated mutant macrophages. These results identify wild-type NLRP3 as the molecular target of MCC950/CRID3 and show that CAPS-related NLRP3 mutants escape efficient MCC950/CRID3 inhibition. Collectively, this work suggests that MCC950/CRID3-based therapies may effectively treat inflammation driven by wild-type NLRP3 but not CAPS-associated mutants.

## Abstract 2

The diarylsulfonylurea-based Cytokine Release Inhibitory Drugs (CRIDs) potently and selectively inhibit the NLRP3 inflammasome pathway, but their molecular target is unknown. This study identifies the NACHT domain of NLRP3 itself as the molecular target of the CRID MCC950/CRID3 and evaluates the functional impact of disease mutations on inflammasome blockade.

## Introduction

Inflammasomes are a suite of multiprotein complexes that play central roles in innate immune responses through their ability to recruit and activate caspase-1 (Casp1) [1,2]. This cysteine protease cleaves the cytokines interleukin (IL)-1β and IL-18 and drives pyroptosis, a highly inflammatory regulated cell death mode that is induced by cleavage of gasdermin D (GSDMD) [3,4]. Among the different inflammasome pathways, the nucleotide-binding-domain (NBD)–and leucine-rich-repeat (LRR)–containing (NLR) family, pyrin-domain–containing 3 (NLRP3) inflammasome responds to the broadest suite of inflammasome agonists that include diverse pathogen-associated molecular patterns (PAMPs); host-derived danger-associated molecular patterns (DAMPs) such as ATP; protein aggregates and β-fibrils such as β-amyloid; a broad range of environmental insults and ionophores such as nigericin; and medically relevant crystals such as alum, calcium pyrophosphate dihydrate (CPPD), monosodium urate (MSU), silica, and asbestos [1,2]. Moreover, the NLRP3 inflammasome is engaged by gram-negative pathogens, lipopolysaccharides (LPS) that are sensed in the cytosol by the non-canonical NLRP3 inflammasome pathway [5–8]. Through the latter mechanism, cleavage of GSDMD by caspase-11—and its human orthologs caspases 4 and 5—promotes assembly of cytolytic GSDMD pores in the plasma membrane that also activate the NLRP3 inflammasome to drive Casp1-dependent IL-1β and IL-18 maturation [3,5]. Consistent with the chemical diversity of NLRP3 stimuli, activation of the NLRP3 inflammasome is thought to converge on sensing of a secondary messenger or cellular state that is universally induced by NLRP3-activating agents [9].

Aberrant NLRP3 inflammasome activity is thought to contribute to the pathogenesis of many chronic diseases, including inflammatory diseases such as gout and pseudogout; metabolic diseases like atherosclerosis and nonalcoholic fatty liver disease (NAFLD)/nonalcoholic steatohepatitis (NASH); and neurodegenerative diseases like Alzheimer’s disease, Parkinson’s disease, and multiple sclerosis [10,11]. Moreover, gain-of-function mutations in and around the central NAIP, CIITA, HET-E, and TP1 (NACHT) domain of NLRP3 cause three autosomal dominantly inherited periodic fever syndromes that together are known as cryopyrin-associated periodic syndrome (CAPS). Symptoms span a clinical spectrum, with Familial Cold Autoinflammatory Syndrome (FCAS) being the mildest; Muckle–Wells syndrome (MWS) being of moderate severity; and Neonatal Onset Multisystem Inflammatory Disease (NOMID)/Chronic Infantile Neurological, Cutaneous, and Articular Syndrome (CINCA) being the most severe form of CAPS, featuring systemic inflammation, neurological and sensory impairment, and deforming arthropathy [12].

Selective and potent inhibitors of the NLRP3 inflammasome may have broad therapeutic potential in CAPS and other diseases [10,11]. Early studies with the sulfonylurea drug glyburide provided proof of concept that PAMP-, DAMP-, and crystal-induced activation of the NLRP3 inflammasome pathway may be selectively targeted without interfering with other inflammasome pathways [13]. The related diarylsulfonylurea compound MCC950/Cytokine Release Inhibitory Drug 3 (CRID3) was originally reported as an inhibitor of IL-1β secretion [14] and subsequently shown to potently and selectively inhibit the NLRP3 inflammasome pathway in murine and human macrophages and monocytes with half maximal inhibitory concentration (IC_50_) values in the low nM range [15,16]. There is significant interest in the discovery and development of diarylsulfonylurea CRIDs such as MCC950/CRID3 for the treatment of CAPS and other diseases based on its ability to curb inflammatory pathology in mouse models of CAPS, the experimental autoimmune encephalomyelitis mouse model of multiple sclerosis, NAFLD/NASH, and many other inflammatory disease models [10,11]. However, the direct molecular target of MCC950/CRID3 in the NLRP3 inflammasome pathway has remained elusive, hampering the rational optimization and development of MCC950/CRID3-based therapies.

Utilizing two different chemoproteomic strategies, we here demonstrate that the NACHT domain of NLRP3 is the molecular target of diarylsulfonylurea CRIDs. Interestingly, we find photoaffinity labeling (PAL) of the NACHT domain of NLRP3 requires an intact (d)ATP-binding pocket and is substantially reduced for most CAPS-associated NLRP3 mutants. In accordance, NLRP3-driven inflammatory pathology in mouse models of CAPS was not efficiently curbed by MCC950/CRID3. Consistently, MCC950/CRID3 abolished circulating levels of IL-1β and IL-18 in LPS-challenged wild-type mice but not in CAPS mice and ex vivo-stimulated mutant macrophages. These results identify the central NACHT domain of wild-type NLRP3 as the molecular target of MCC950/CRID3 and show that CAPS-related NACHT mutations prevent efficient MCC950/CRID3 inhibition. Collectively, this work suggests that MCC950/CRID3-based therapies may effectively treat inflammation driven by wild-type NLRP3 but not CAPS-associated mutants.

## Results

### MCC950/CRID3 selectively binds to human and murine NLRP3

**Fig 1:**
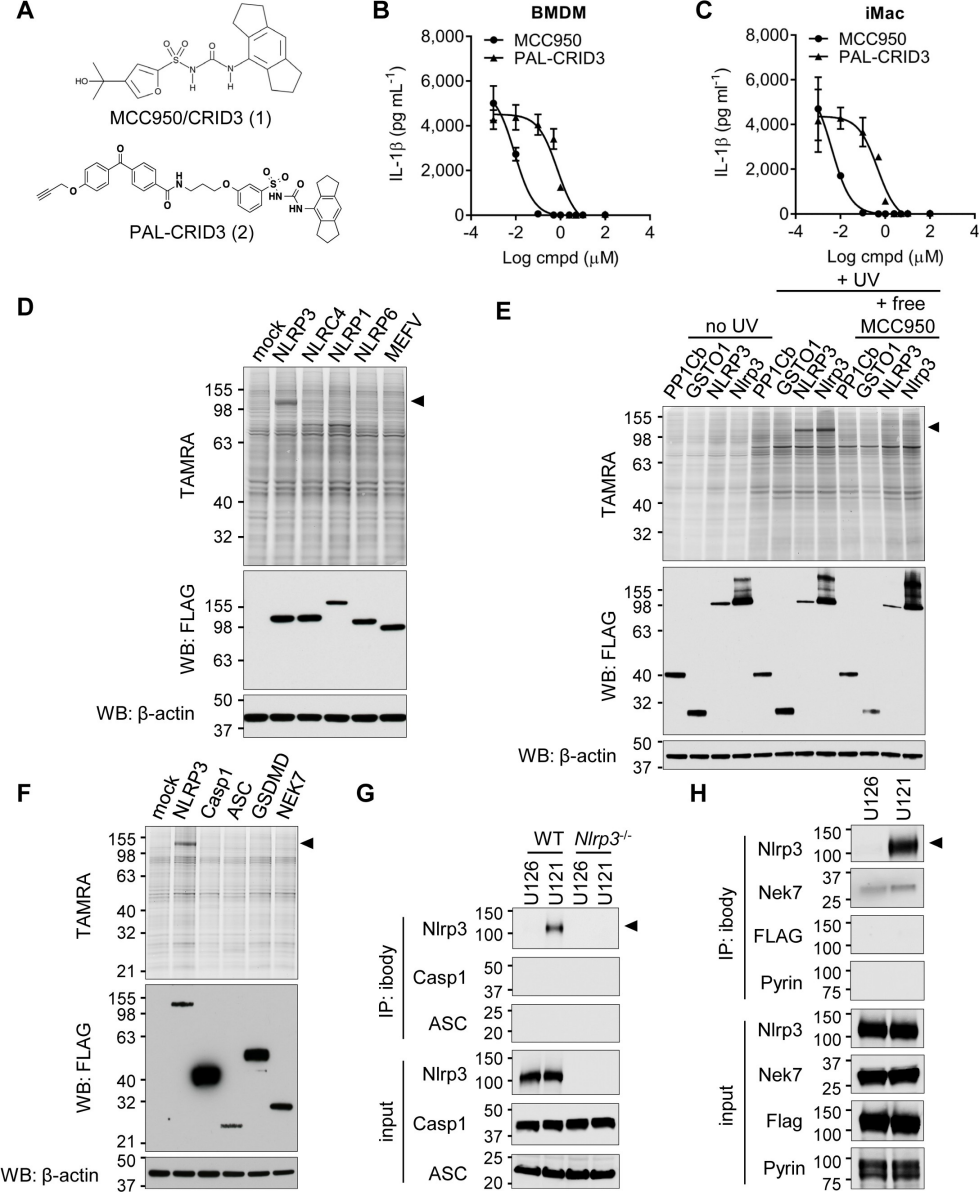
MCC950/CRID3 directly targets NLRP3. (A) Chemical structure of MCC950/CRID3 and the photoaffinity probe PAL-CRID3. (B–C) LPS-primed BMDMs (B) and iMac (C) were stimulated with Nig in presence of the indicated concentrations of MCC950/CRID3 or PAL-CRID3. Supernatants were analyzed for IL-1β secretion. BMDM: IC50_MCC950_ = 0.009 μM; IC50_PAL-CRID3_ = 0.731 μM; iMac: IC50_MCC950_ = 0.004 μM; IC50_PAL-CRID3_ = 0.453 μM. (D) HEK293T cells transfected with the indicated plasmids were incubated with PAL-CRID3 and subsequently irradiated. After cell lysis and click chemistry conjugation, lysates were assayed by TAMRA imaging and by immunoblot analysis with the indicated antibodies. (E) HEK293T cells transfected with the indicated plasmids were incubated with PAL-CRID3 in the absence or presence of free MCC950/CRID3 and subsequently irradiated (+ UV) or left untreated (no UV). After cells are lysed, click chemistry is performed, and lysates were assayed by TAMRA imaging and by immunoblot analysis with the indicated antibodies. (F) HEK293T cells transfected with the indicated plasmids were incubated with PAL-CRID3 and subsequently irradiated. After cells are lysed, click chemistry is performed, and lysates are assayed by TAMRA imaging and by immunoblot analysis with the indicated antibodies. (G) Lysates of LPS-primed wild-type or Nlrp3-deficient BMDMs were incubated with iBody U126 (Ctrl) or iBody U121 (MCC950/CRID3) and analyzed by pulldown assay with streptavidin agarose. Precipitates and lysates were assayed by immunoblot analysis with the indicated antibodies. (H) Lysates of LPS-primed Nlrc4^Flag/Flag^ BMDMs were incubated with iBody U126 (Ctrl) or iBody U121 (MCC950/CRID3), and lysates were analyzed by pulldown assay with streptavidin agarose. Precipitates and lysates were assayed by immunoblot analysis with the indicated antibodies. The numerical values underlying Fig 1B and 1C can be found in S1 Data. Graphs show mean ± SD of triplicate wells and represent three independent experiments. ASC, apoptosis-associated speck-like protein; BMDM, bone-marrow–derived macrophage; Casp1, caspase-1; cmpd, compound; CRID, Cytokine Release Inhibitory Drug; Ctrl, control; ER-Hoxb8, estrogen-regulated homeobox protein Hox-B8; GSDMD, gasdermin D; GSTO1, Glutathione S-transferase omega-1; HEK, human embryonic kidney; IC_50_, half maximal inhibitory concentration; IL, interleukin; iMac, ER-Hoxb8–immortalized macrophages; LPS, lipopolysaccharides; LRR, leucine-rich repeat; MEFV, Mediterranean fever; NBD, nucleotide-binding domain; NEK7, never-in-mitosis-gene-a–related kinase 7; Nig, nigericin; NLR, NBD- and LRR-containing; Nlrc4, NLR family, caspase-recruitment-domain–containing 4; NLRP3, NLR family, pyrin-domain–containing 3; PAL, photoaffinity labeling; PP1Cb, protein phosphatase 1 catalytic subunit β isoform; TAMRA, 5-carboxytetramethylrhodamine; WB, western blot.

As a first approach to identify the molecular target of MCC950/CRID3, we made use of PAL in combination with click chemistry [17]. Guided by limited structure-activity studies, a cell-permeable photoaffinity probe was synthesized (Fig 1A, compound PAL-CRID3) that contains a photoreactive benzophenone group to enable direct covalent labeling of MCC950/CRID3 targets upon exposure to UV light. The alkyne functionality in PAL-CRID3 allowed in situ click reaction with a 5-carboxytetramethylrhodamine (TAMRA) fluorescent reporter to support in-gel fluorescence detection of the covalent MCC950/CRID3-protein adduct by SDS-PAGE. A dose–response analysis confirmed that PAL-CRID3 retained the ability to potently inhibit NLRP3 inflammasome activation with submicromolar IC_50_ values. PAL-CRID3 inhibited nigericin-induced IL-1β secretion from N-palmitoyl-S-dipalmitoylglyceryl Cys-Ser-(Lys)4 (Pam3CSK4)-primed primary bone-marrow–derived macrophages (BMDMs) (Fig 1B and S1 Data; IC_50_ = 731 nM) and estrogen-regulated homeobox protein Hox-B8 (ER-Hoxb8)–immortalized macrophages (iMac) (Fig 1C and S1 Data; IC_50_ = 453 nM). As a reference, MCC950/CRID3 inhibited nigericin-induced IL-1β secretion with IC_50_ values of 9 and 4 nM, respectively (Fig 1B and 1C; S1 Data). We hypothesize that the lesser activity of PAL-CRID3 compared to MCC950/CRID3 is a combination of physical property and binding differences that decrease target occupancy.

To screen candidate targets of MCC950/CRID3, HEK293T cells overexpressing Flag-epitope–tagged fusions of the human inflammasome sensor proteins NLRP3, NLRP1, NLRC4, NLRP6, and MEFV were incubated with PAL-CRID3 and exposed to UV light to allow covalent binding of PAL-CRID3 to potential targets. Following cell lysis, the probe was conjugated to the TAMRA reporter by click chemistry, and protein lysates were separated by SDS-PAGE. Notably, in-gel fluorescence imaging showed significant TAMRA labeling of ectopically expressed NLRP3 but not other inflammasome sensors in the panel (Fig 1D). To further extend these findings, we next confirmed binding to murine Nlrp3 (Fig 1E). As controls, protein phosphatase PP1Cb and glutathione S-transferase GSTO1, which have been proposed as the target of CRID compounds [14], were not labeled by PAL-CRID3 (Fig 1E). Moreover, binding of PAL-CRID3 to human NLRP3 and murine Nlrp3 was only observed following UV cross-linking, and labeling was rescued by competition with excess MCC950/CRID3 (Fig 1E), thus ruling out nonspecific cross-linking and validating the specificity of these findings. To further characterize the interaction of PAL-CRID3 with components of the NLRP3 inflammasome, we assessed binding to human never-in-mitosis-gene-a–related kinase 7 (NEK7), apoptosis-associated speck-like protein (ASC), Casp1, and GSDMD. PAL-CRID3 failed to label the NLRP3 inflammasome components in the panel apart from NLRP3 (Fig 1F). Collectively, these results suggest that CRID compounds, including MCC950/CRID3, inhibit NLRP3 inflammasome signaling by directly binding to NLRP3.

To confirm and extend these findings to endogenous NLRP3, we made use of recently described iBody technology to immobilize MCC950/CRID3 on polymers that enabled immunoprecipitation of MCC950/CRID3 targets [18]. In brief, MCC950/CRID3 was stochastically modified with a photoactivatable phenyldiazirine linker, and the resulting isomeric mixture was conjugated to a hydrophilic *N*-(2-hydroxypropyl)methacrylamide (HPMA) polymer backbone that is decorated with a biotin affinity tag (for details, please refer to the Materials and Methods section and [18]). The MCC950/CRID3 iBody conjugate is further referred to as iBody U121. The corresponding iBody conjugate lacking MCC950/CRID3 served as a negative control and is referred to as iBody U126.

BMDMs of wild-type (C57BL/6J) and *Nlrp3*^*−/−*^ mice were primed with LPS to transcriptionally up-regulate NLRP3 inflammasome components [19], and cell lysates were subsequently incubated with iBody U121 (MCC950/CRID3) or iBody U126 (control), followed by immunoprecipitation with streptavidin-coupled beads. Contrary to control iBody U126, iBody U121 immunoprecipitated endogenous Nlrp3 from wild-type BMDMs (Fig 1G). iBody U121 failed to pull down inflammasome components ASC and Casp1 (Fig 1G). As expected, the immunoreactive band was absent from immunoprecipitates and lysates of LPS-primed *Nlrp3*-deficient macrophages (Fig 1G). Given the lack of suitable antibodies to detect endogenous NLRC4, we made use of reported *Nlrc4*^3×Flag/3×Flag^ knock-in mice [20,21] to further validate selective targeting of NLRP3. Consistent with our earlier results, only iBody U121 precipitated endogenous Nlrp3 from LPS-primed *Nlrc4*^3×Flag/3×Flag^ BMDMs (Fig 1H). Comparable background binding of Nek7 was observed with iBody U121 and control iBody U126, whereas neither Nlrc4 (detected using FLAG antibodies), Nek7, nor Pyrin were retrieved in iBody U121 immunoprecipates of LPS-primed *Nlrc4*^Flag/Flag^ BMDMs (Fig 1H). Collectively, these results show that MCC950/CRID3 selectively binds to Nlrp3 in LPS-primed macrophages.

### PAL-CRID3 targets the central NACHT domain of wild-type NLRP3 but not CAPS mutants

**Fig 2:**
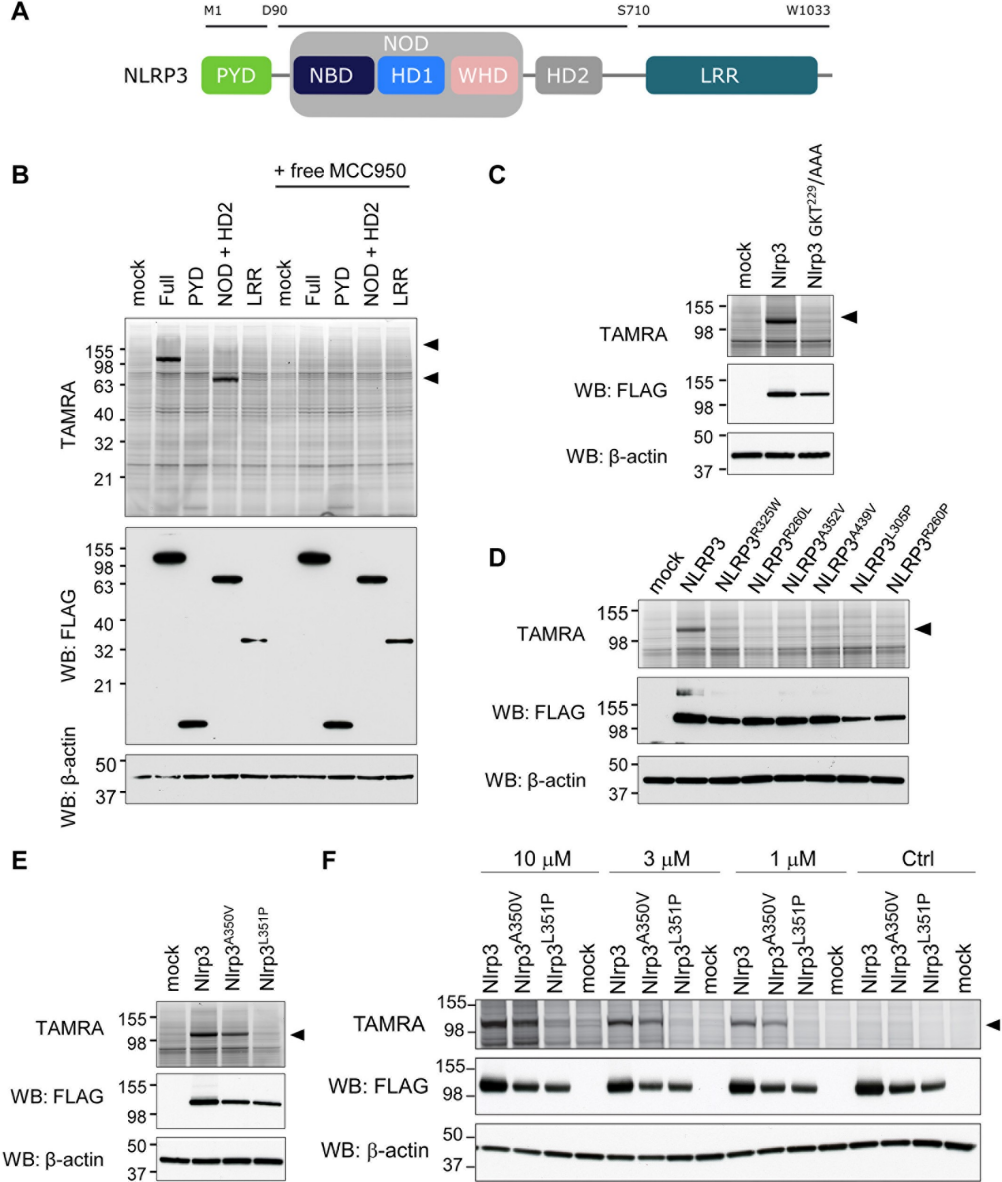
PAL-CRID3 binding requires an intact (d)ATP-binding pocket and fails to bind CAPS-associated NLRP3 mutants. (A) Schematic diagram of NLRP3 depicting the different domains used in this study. (B) HEK293T cells transfected with the indicated plasmids were incubated with PAL-CRID3 in the absence or presence of free MCC950/CRID3 and subsequently irradiated. After cells are lysed, click chemistry is performed, and lysates are assayed by TAMRA imaging and by immunoblot analysis with the indicated antibodies. (C–F) HEK293T cells were transfected with the indicated plasmids and incubated with 1 μM PAL-CRID3, unless indicated otherwise, and subsequently irradiated. After cell lysis, click chemistry was performed, and lysates were assayed by TAMRA imaging and by immunoblot analysis with the indicated antibodies. CAPS, cryopryin-associated periodic syndrome; CRID, Cytokine Release Inhibitory Drug; HD, helical domain; HEK, human embryonic kidney; LRR, leucine-rich repeat; NBD, nucleotide-binding domain; NLR, NBD- and LRR-containing; NLRP3, NLR family, pyrin-domain–containing 3; NOD, nucleotide-binding oligomerization domain; PAL, photoaffinity labeling; PYD, Pyrin domain; TAMRA, 5-carboxytetramethylrhodamine; WB, western blot; WHD, winged helix domain.

NLRP3 consists of an amino-terminal Pyrin domain (PYD), a central NACHT domain, and carboxy-terminal LRRs that are thought to lock NLRP3 in an inactive conformation. To map the NLRP3 region(s) to which MCC950/CRID3 binds, we generated deletion mutants that cover the following three regions of human NLRP3: (i) the N-terminal PYD; (ii) the central NACHT domain (comprising the nucleotide-binding oligomerization domain (NOD) and helical domain [HD] 2); and (iii) the carboxy-terminal LRR region (Fig 2A). Consistent with our previous results (Fig 1), TAMRA analysis showed significant binding of PAL-CRID3 to ectopically expressed full-length NLRP3 in HEK293T cells (Fig 2B). In addition, we observed binding of PAL-CRID3 to the isolated NACHT (NOD + HD2) region of NLRP3 but not to the PYD and LRR domains (Fig 2B). Labeling of both full-length NLRP3 and the isolated NACHT region by PAL-CRID3 was rescued by treatment with an excess amount of free MCC950/CRID3 (Fig 2B), confirming specificity and suggesting PAL-CRID3 and MCC950/CRID3 compete for the same binding site. Binding of ATP/dATP to the Walker A pocket in the NACHT domain is essential for NLRP3 inflammasome assembly and function [22]. Interestingly, mutation of the Walker A motif (GKT^229^/AAA) in full-length Nlrp3 abolished PAL-CRID3 binding (Fig 2C), suggesting that an intact ATP-binding pocket is required for strongest binding of diarylsulfonylurea CRID compounds.

The central NACHT domain of NLRP3 also contains most reported gain-of-function mutations that cause CAPS disease (https://infevers.umai-montpellier.fr/web/index.php). This prompted us to explore PAL-CRID3 binding to CAPS-associated NLRP3 mutants. Unexpectedly, introducing a random selection of 6 different CAPS mutations in the NACHT domain of human NLRP3 that are associated with, respectively, MWS (NLRP3^R325W^, NLRP3^R260L^, NLRP3^A352V^), FCAS (NLRP3^A439V^, NLRP3^L305P^), or NOMID (NLRP3^R260P^) all blunted binding of PAL-CRID3 to full-length human NLRP3 (Fig 2D). The classical MWS A352V and FCAS L353P mutations in human NLRP3 correspond to the A350V and L351P mutations that have been knocked into the murine *Nlrp3* gene to model CAPS disease in mice [23]. Notably, the Nlrp3^A350V^ mutant did not significantly affect labeling by PAL-CRID3, whereas the Nlrp3^L351P^ mutation abolished labeling by PAL-CRID3 at 1 μM (Fig 2E). A dose–response analysis showed that binding to the Nlrp3^L351P^ mutant was also substantially affected with higher (3 μM and 10 μM) PAL-CRID3 concentrations (Fig 2F). Together, these results suggest that PAL of the NACHT domain requires an intact (d)ATP-binding pocket and is substantially reduced for most CAPS-associated NLRP3 mutants.

### MCC950/CRID3 inhibits the inflammasome in wild-type but not *Nlrp3*^L351P^ macrophages

Considering our observation that CAPS-associated Nlrp3 mutants escape PAL-CRID3 binding may have potential implications for treating CAPS with MCC950/CRID3, we sought to functionally validate these results with MCC950/CRID3 in the reported *Nlrp3*^L351P^ CAPS model [23]. To this end, mice that were homozygous for the *Nlrp3*^L351P^ allele were bred to transgenic mice that hemizygously expressed the tamoxifen-inducible Cre recombinase-estrogen receptor (Cre-ERT2) fusion gene (CreT) [24]. Following tamoxifen treatment and excision of the floxed neomycin resistance cassette, BMDMs of the resulting *Nlrp3*^L351P/+^CreT^+^ mice expressed Nlrp3 from both the wild-type and *Nlrp3*^L351P^ alleles. Macrophages from Cre-ERT2–negative littermates (*Nlrp3*^L351P/+^CreT^−^), which only express Nlrp3 from the wild-type allele, were used as controls in these experiments.

**Fig 3:**
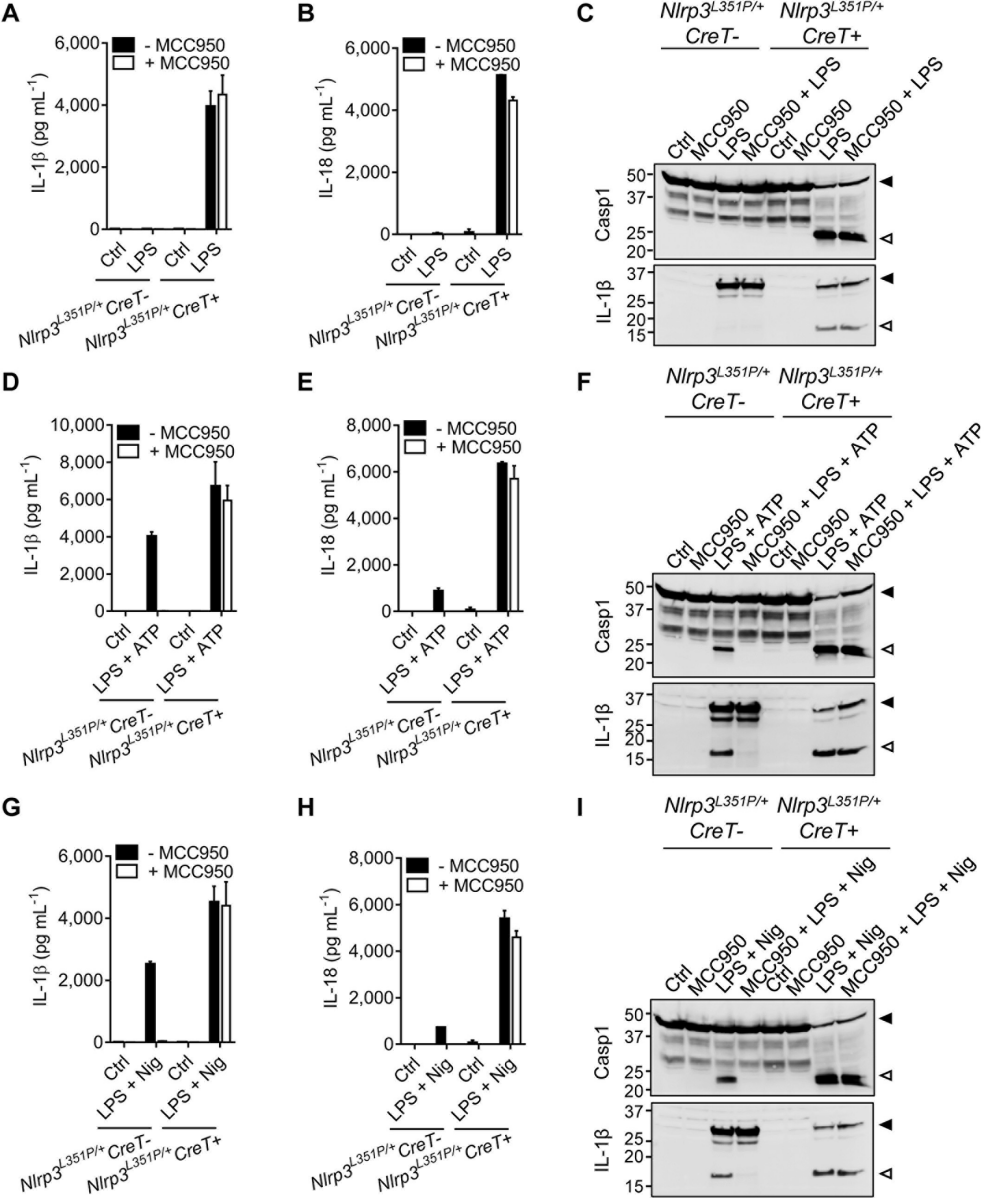
MCC950/CRID3 inhibits the inflammasome in wild-type but not Nlrp3^L351P^ macrophages. (A–C) BMDMs from tamoxifen-treated *Nlrp3*^*L351P/+*^*CreT−* and *Nlrp3*^*L351P/+*^ *CreT+* mice were left untreated (Ctrl) or treated with LPS in absence or presence of MCC950/CRID3. Supernatants were analyzed for IL-1β (A) and IL-18 (B) secretion and lysates were immunoblotted for Casp1 and IL-1β (C). (D–F) BMDMs from tamoxifen-treated *Nlrp3*^*L351P/+*^*CreT−* and *Nlrp3*^*L351P/+*^*CreT+* mice were left untreated (Ctrl) or primed with LPS and stimulated with ATP in absence or presence of MCC950/CRID3. Supernatants were analyzed for IL-1β (D) and IL-18 (E) secretion and lysates were immunoblotted for Casp1 and IL-1β (F). (G–I) BMDMs from tamoxifen-treated *Nlrp3*^*L351P/+*^*CreT−* and *Nlrp3*^*L351P/+*^*CreT+* mice were left untreated (Ctrl) or primed with LPS and stimulated with Nig in absence or presence of MCC950/CRID3. Supernatants were analyzed for IL-1β (G) and IL-18 (H) secretion, and lysates were immunoblotted for Casp1 and IL-1β (I). The numerical values underlying Fig 3A, 3B, 3D, 3E, 3G, and 3H can be found in S2 Data. Graphs show mean ± SD of triplicate wells and represent three independent experiments. BMDM, bone-marrow–derived macrophage; Casp1, caspase-1; Cre-ERT2, Cre recombinase-estrogen receptor fusion protein; CreT, Cre-ERT2 fusion gene; CRID, Cytokine Release Inhibitory Drug; Ctrl, control; IL, interleukin; LPS, lipopolysaccharides; LRR, leucine-rich repeat; NBD, nucleotide-binding domain; Nig, nigericin; NLR, NBD- and LRR-containing; NLRP3, NLR family, pyrin-domain–containing 3.

Culture media of LPS-stimulated *Nlrp3*^L351P/+^CreT^+^ macrophages contained significant levels of IL-1β (Fig 3A and S2 Data) and IL-18 (Fig 3B and S2 Data), which were associated with marked maturation of Casp1 and IL-1β in cell lysates (Fig 3C). As reported [23], these LPS-induced inflammasome responses were driven by the CAPS-associated *Nlrp3*^L351P^ allele because *Nlrp3*^L351P/+^CreT^−^ BMDMs failed to secrete detectable levels of IL-1β (Fig 3A and S2 Data) and IL-18 (Fig 3B and S2 Data) and did not contain mature Casp1 and IL-1β in their cell lysates (Fig 3C). Consistent with our previous results that CAPS-associated Nlrp3^L351P^ escaped PAL-CRID3 binding, we found that MCC950/CRID3 failed to inhibit the above inflammasome responses driven by the *Nlrp3*^L351P^ allele. We confirmed that MCC950/CRID3 was active against wild-type NLRP3 because it abolished levels of LPS+ATP–induced secretion of IL-1β (Fig 3D and S2 Data) and IL-18 (Fig 3E and S2 Data) in culture media of *Nlrp3*^L351P/+^CreT^−^ BMDMs (which are solely driven by wild-type Nlrp3 in this genotype, given the absence of a Cre-ERT2 transgene), as well as the concomitant maturation of Casp1 and IL-1β in the corresponding cell lysates (Fig 3F). Similarly, MCC950/CRID3 abolished LPS + nigericin-induced secretion of IL-1β (Fig 3G and S2 Data) and IL-18 (Fig 3H and S2 Data) and maturation of Casp1 and IL-1β by *Nlrp3*^L351P/+^CreT^−^ macrophages (Fig 3I). In marked contrast, LPS + ATP- and LPS + nigericin-induced inflammasome responses were insensitive to MCC950/CRID3 blockade in *Nlrp3*^L351P/+^CreT^+^ BMDMs that express both the *Nlrp3*^L351P^ allele and wild-type Nlrp3 (Fig 3D–3I and S2 Data). Together, these results establish that the CAPS-associated *Nlrp3*^L351P^ allele is insensitive to MCC950/CRID3 inhibition.

### MCC950/CRID3 inhibition of inflammasome responses in *Nlrp3*^A350V^ macrophages

Unlike for Nlrp3^L351P^, labeling of Nlrp3^A350V^ by PAL-CRID3 was not significantly impacted by the mutation (Fig 2E and 2F and S3 Data). To address whether this was mirrored by potent inhibition of *Nlrp3*^A350V^-driven inflammasome responses by MCC950/CRID3, mice that were homozygous for the previously reported *Nlrp3*^A350V^ allele were bred to the CreT transgenic mice described above. Following tamoxifen treatment and excision of the floxed neomycin resistance cassette, BMDMs of the resulting Nlrp3^A350V/+^CreT^+^ mice expressed Nlrp3 from both the wild-type and *Nlrp3*^A350V^ alleles. BMDMs from CreT-negative littermates (*Nlrp3*^A350V/+^CreT^−^), which only express Nlrp3 from the wild-type allele, were used as controls in these experiments.

**Fig 4:**
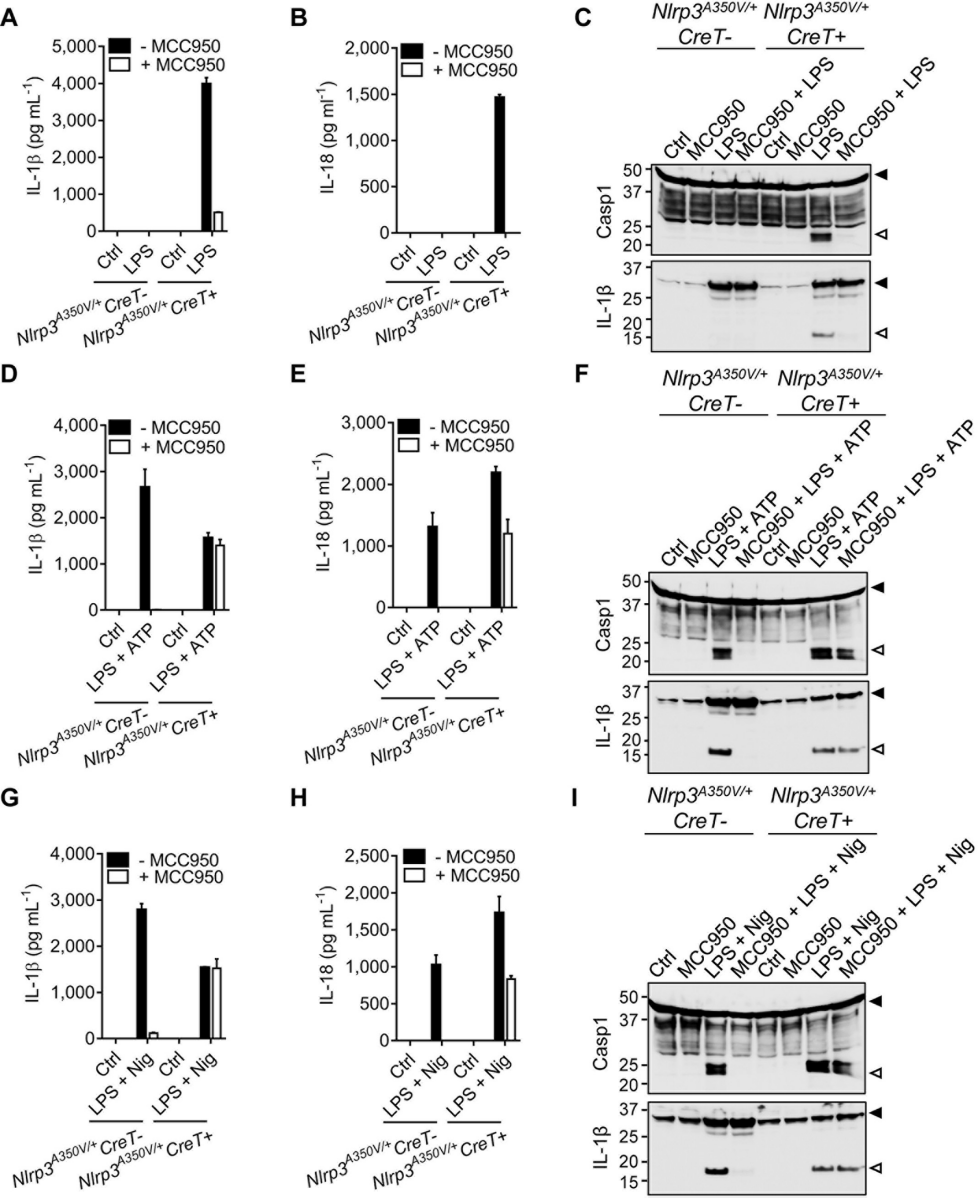
MCC950/CRID3 inhibition of inflammasome responses in Nlrp3^A350V^ macrophages. (A–C) BMDMs from tamoxifen-treated *Nlrp3*^*A350V/+*^*CreT−* and *Nlrp3*^*A350V/+*^*CreT+* mice were left untreated (Ctrl) or treated with LPS in absence or presence of MCC950/CRID3. Supernatants were analyzed for IL-1β (A) and IL-18 (B) secretion, and lysates were immunoblotted for Casp1 and IL-1β (C). (D–F) BMDMs from tamoxifen-treated *Nlrp3*^*A350V/+*^*CreT−* and *Nlrp3*^*A350V/+*^*CreT+* mice were left untreated (Ctrl) or primed with LPS and stimulated with ATP in absence or presence of MCC950/CRID3. Supernatants were analyzed for IL-1β (D) and IL-18 (E) secretion, and lysates were immunoblotted for Casp1 and IL-1β (F). (G–I) BMDMs from tamoxifen-treated *Nlrp3*^*A350V/+*^*CreT−* and *Nlrp3*^*A350V/+*^*CreT+* mice were left untreated (Ctrl) or primed with LPS and stimulated with Nig in absence or presence of MCC950/CRID3. Supernatants were analyzed for IL-1β (G) and IL-18 (H) secretion, and lysates were immunoblotted for Casp1 and IL-1β (I). The numerical values underlying Fig 4A, 4B, 4D, 4E, 4G, and 4H can be found in S3 Data. Graphs show mean ± SD of triplicate wells and represent three independent experiments. BMDM, bone-marrow–derived macrophage; Casp1, caspase-1; Cre-ERT2, Cre recombinase-estrogen receptor fusion protein; CreT, Cre-ERT2 fusion gene; CRID, Cytokine Release Inhibitory Drug; Ctrl, control; IL, interleukin; LPS, lipopolysaccharides; LRR, leucine-rich repeat; NBD, nucleotide-binding domain; Nig, nigericin; NLR, NBD- and LRR-containing; NLRP3, NLR family, pyrin-domain–containing 3.

Like *Nlrp3*^L351P/+^CreT^+^ BMDMs (Fig 3A–3C and S2 Data), *Nlrp3*^A350V/+^CreT^+^ macrophages that expressed Nlrp3^A350V^ secreted high levels of IL-1β (Fig 4A and S3 Data) and IL-18 (Fig 4B and S3 Data) in response to LPS stimulation alone. This was accompanied by proteolytic maturation of Casp1 and proIL-1β, as demonstrated by immunoblot analysis (Fig 4C). As expected, these responses were absent from LPS-stimulated *Nlrp3*^A350V/+^CreT^−^ BMDMs that expressed wild-type Nlrp3 only (Fig 4A–4C and S3 Data). MCC950/CRID3 potently inhibited LPS-induced IL-1β (Fig 4A and S3 Data) and IL-18 (Fig 4B and S3 Data) secretion and Nlrp3^A350V^-driven cleavage of Casp1 and IL-1β (Fig 4C and S3 Data), in marked contrast to *Nlrp3*^L351P/+^CreT^+^ macrophages that proved insensitive to MCC950/CRID3 blockade (Fig 3A–3C and S2 Data).

LPS + ATP and LPS + nigericin potently triggered IL-1β and IL-18 secretion and maturation of Casp1 and pro-IL-1β from both *Nlrp3*^A350V/+^CreT^+^ and *Nlrp3*^A350V/+^CreT^−^ control BMDMs (Fig 4D–4I and S3 Data). However, whereas MCC950/CRID3 abolished LPS + ATP- and LPS + nigericin-induced IL-1β secretion from *Nlrp3*^A350V/+^CreT^−^ control BMDMs, it failed to alter secretion of IL-1β from parallelly treated *Nlrp3*^A350V/+^CreT^+^ BMDMs (Fig 4D and 4G; S3 Data). MCC950/CRID3 also abrogated LPS + ATP- and LPS + nigericin-induced IL-18 secretion from control *Nlrp3*^A350V/+^CreT^−^ BMDMs but only reduced IL-18 secretion from parallelly stimulated *Nlrp3*^A350V/+^CreT^+^ macrophages by about 50% (Fig 4E and 4H; S3 Data). Aligned with these results, MCC950/CRID3 inhibited LPS + ATP- and LPS + nigericin-induced cleavage of Casp1 and pro-IL-1β in control *Nlrp3*^A350V/+^CreT^−^ BMDMs but not in *Nlrp3*^A350V/+^CreT^+^ macrophages (Fig 4F and 4I). Together, these results show that although binding of PAL-CRID3 to Nlrp3^A350V^ was not significantly compromised, the mutation subtly alters the ability of MCC950/CRID3 to inhibit inflammasome activation in primary macrophages, with potent inhibition seen only in response to LPS but not following “signal 2” triggers such as ATP and nigericin.

### Inflammasome inhibition in homozygous mutant macrophages

The studies described above were performed in heterozygous macrophages that express both wild-type and CAPS-associated Nlrp3 mutants. Considering that the Nlrp3 NACHT region facilitates Nlrp3 oligomerization, we decided to further assess MCC950/CRID3 responses in macrophages that uniquely express the CAPS-associated Nlrp3 mutants in the absence of wild-type Nlrp3. To do so, we transduced wild-type, *Nlrp3*^A350V/A350V^, and *Nlrp3*^L351P/L351P^ BMDMs with Cre-recombinase–expressing lentiviruses to excise the neomycin resistance cassette that is placed upstream of the Nlrp3 mutation and to allow expression of the CAPS-associated Nlrp3 mutants.

**Fig 5:**
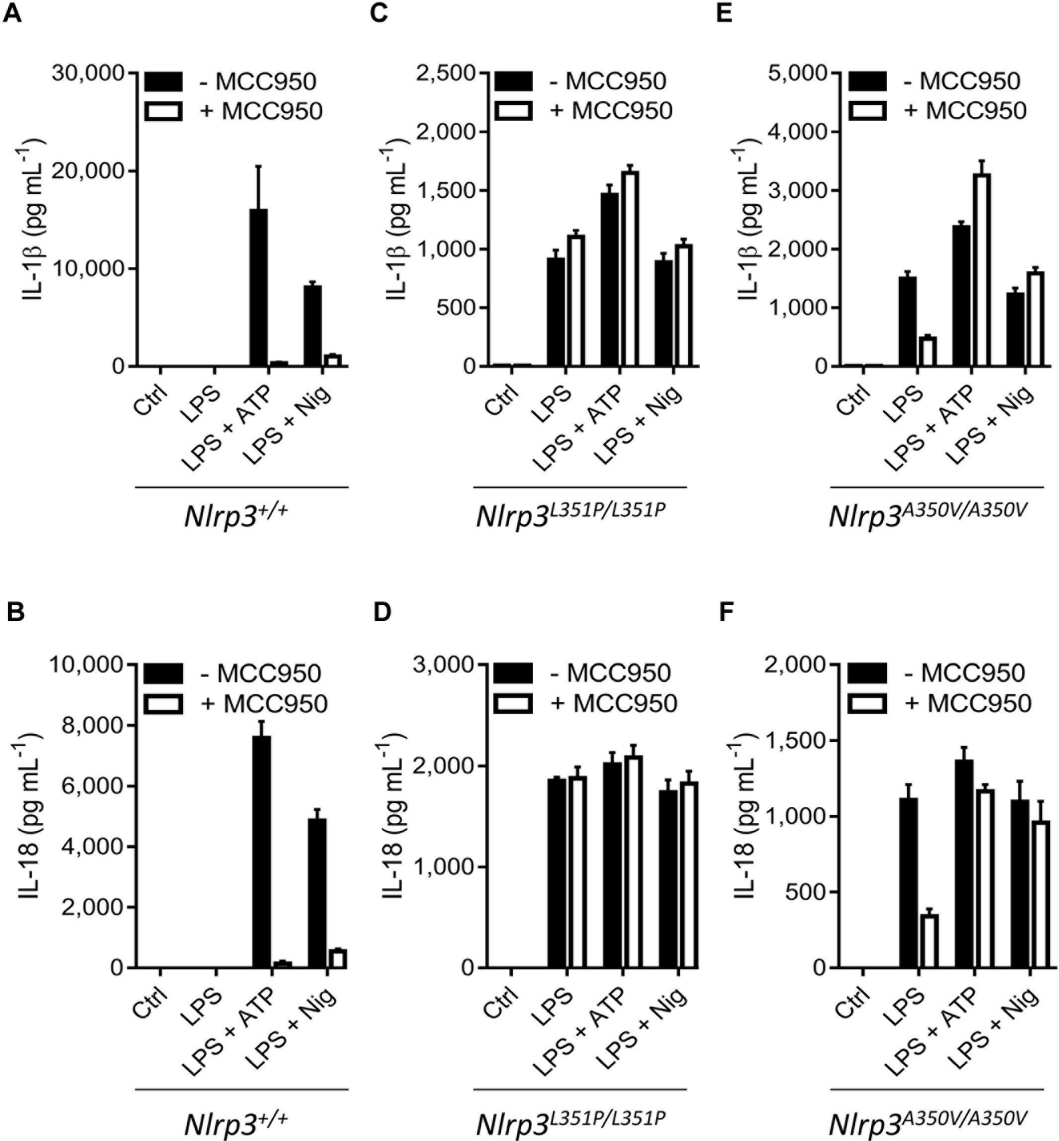
Inflammasome inhibition in homozygous mutant macrophages. (A–F) Wild-type (A–B), *Nlrp3*^*L351P/L351P*^ (C–D), or *Nlrp3*^*A350V/A350V*^ (E–F) BMDMs that were transduced with a Cre-recombinase–expressing lentiviral vector were left unstimulated (Ctrl) or stimulated with LPS and then left untreated or treated with ATP or Nig in absence or presence of MCC950/CRID3. Supernatants were analyzed for IL-1β (A,C,E) and IL-18 (B,D,F) secretion. The numerical values underlying Fig 5A–5F can be found in S4 Data. Graphs show mean ± SD of triplicate wells and represent three independent experiments. BMDM, bone-marrow–derived macrophage; Cre, Cre recombinase; CRID, Cytokine Release Inhibitory Drug; Ctrl, control; IL, interleukin; LPS, lipopolysaccharides; LRR, leucine-rich repeat; NBD, nucleotide-binding domain; Nig, nigericin; NLR, NBD- and LRR-containing; NLRP3, NLR family, pyrin-domain–containing 3.

As expected, wild-type macrophages failed to secrete IL-1β and IL-18 in response to LPS alone, and MCC950/CRID3 abolished secretion of IL-1β and IL-18 when wild-type BMDMs were stimulated with LPS + ATP or LPS + nigericin (Fig 5A and 5B; S4 Data). LPS stimulation alone was sufficient to induce extracellular release of IL-1β and IL-18 from homozygous *Nlrp3*^L351P/L351P^ and *Nlrp3*^A350V/A350V^ macrophages (Fig 5C–5F; S4 Data). MCC950/CRID3 inhibited Nlrp3^L351P^-induced secretion of IL-1β and IL-18 neither in response to LPS alone nor when combined with “signal 2” agents ATP or nigericin (Fig 5C and 5D; S4 Data), unequivocally establishing that Nlrp3^L351P^-induced inflammasome activation is insensitive to MCC950/CRID3 blockade. Consistent with our previous results in heterozygous *Nlrp3*^A350V^ mutant macrophages, MCC950/CRID3 partially inhibited LPS-induced IL-1β and IL-18 levels in culture supernatants of homozygous *Nlrp3*^A350V/A350V^ macrophages, but this was not observed in response to LPS + ATP and LPS + nigericin (Fig 5E and 5F; S4 Data).

### In vivo MCC950/CRID3 inhibition of inflammasome activation in CAPS disease models

**Fig 6:**
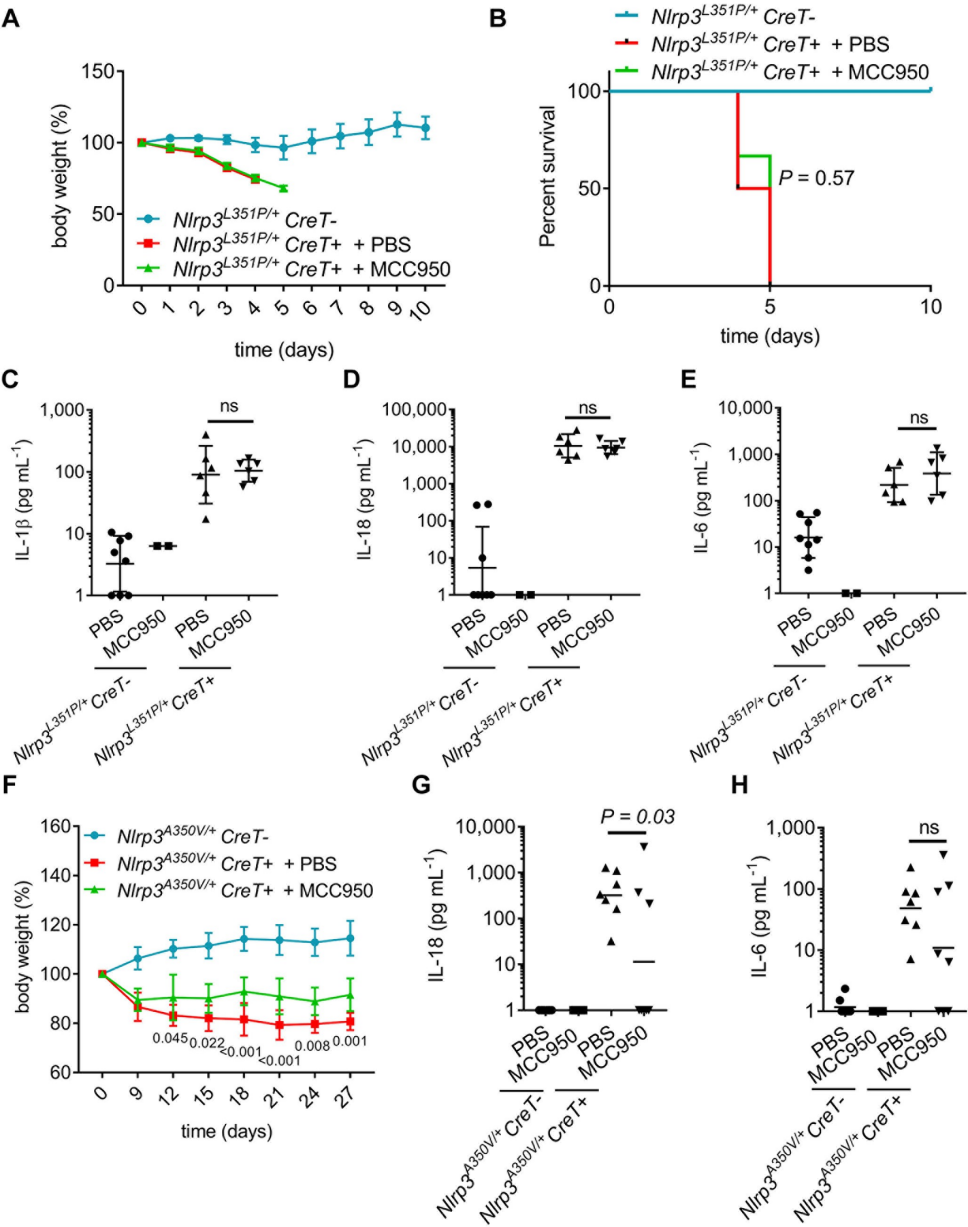
In vivo MCC950/CRID3 inhibition of inflammasome activation in CAPS disease models. (A–B) Growth (A) and survival (B) curves of tamoxifen-treated *Nlrp3*^*L351P/+*^*CreT+* that received daily injections of PBS (*n* = 6) or 50 mg kg^−1^ MCC950/CRID3 (*n* = 6) and of tamoxifen-treated *CreT−* littermates (*n* = 6). (C–E) IL-1β (C), IL-18 (D), and IL-6 (E) cytokine levels in serum obtained at day 4 from tamoxifen-treated *Nlrp3*^*A350V/+*^*CreT+* mice that received daily injections of PBS (n = 6) or MCC950/CRID3 (n = 6). Serum of tamoxifen-treated *CreT−* littermates injected with PBS (*n* = 8) or MCC950/CRID3 (*n* = 2) was used as control. (F) Growth curves of tamoxifen-treated *Nlrp3*^*A350V/+*^*CreT+* that received daily injections of PBS (*n* = 6) or 50 mg kg^−1^ MCC950/CRID3 (*n* = 7) and of tamoxifen-treated *CreT−* littermates (*n* = 6). (G–H) IL-18 (G) and IL-6 (H) cytokine analysis of serum obtained at day 4 from tamoxifen-treated *Nlrp3*^*A350V/+*^*CreT+* mice that received daily injections of PBS (*n* = 7) or MCC950/CRID3 (*n* = 8). Serum of tamoxifen-treated *CreT−* littermates treated with PBS (*n* = 8) or MCC950/CRID3 (*n* = 5) was used as control. The numerical values underlying Fig 6A–6H can be found in S5 Data. Graphs show mean ± SD and represent two independent experiments. CAPS, cryopyrin-associated periodic syndrome; Cre-ERT2, Cre recombinase-estrogen receptor fusion protein; CreT, Cre-ERT2 fusion gene; CRID, Cytokine Release Inhibitory Drug; IL, interleukin; LRR, leucine-rich repeat; NBD, nucleotide-binding domain; NLR, NBD- and LRR-containing; NLRP3, NLR family, pyrin-domain–containing 3; ns, not significant.

Myeloid-specific expression of the *Nlrp3*^L351P^ and *Nlrp3*^A350V^ alleles in knock-in mice was shown to drive systemic inflammation accompanied by, respectively, embryonic and perinatal lethality that in both cases required Nlrp3 inflammasome activation [23]. To seek further validation of the notion that the *Nlrp3*^L351P^ mutations escape MCC950/CRID3 inhibition, we next investigated how MCC950/CRID3 treatment impacts on the CAPS phenotype of *Nlrp3*^L351P/+^CreT^+^ mice. As expected, serum levels of IL-1β, IL-18, and IL-6 were significantly increased in *Nlrp3*^L351P/+^CreT^+^ mice three days after tamoxifen dosing relative to the basal levels of tamoxifen-treated *Nlrp3*^L351P/+^CreT^−^ littermate mice (S1A–S1C Fig and S7 Data). Moreover, tamoxifen treatment resulted in *Nlrp3*^L351P/+^CreT^+^ mice presenting with substantial weight loss and mortality, with all mice being lost or requiring termination because of humane endpoints within 5 days after commencing tamoxifen treatment (Fig 6A and 6B; S5 Data). As a control, tamoxifen administration did not alter body weight or survival of *Nlrp3*^L351P/+^CreT^−^ mice (Fig 6A and 6B; S5 Data). Daily intraperitoneal (IP) injection of MCC950/CRID3 did not rescue body weight loss or mortality rates of *Nlrp3*^L351P/+^CreT^+^ mice, suggesting that MCC950/CRID3 failed to inhibit in vivo Nlrp3^L351P^-induced inflammasome activation (Fig 6A and 6B; S5 Data). In agreement, IP dosing of MCC950/CRID3 failed to reduce tamoxifen-induced levels of IL-1β, IL-18, and IL-6 in serum of *Nlrp3*^L351P/+^CreT^+^ mice (Fig 6C–6E; S5 Data). Thus, the CAPS-associated L351P mutation renders Nlrp3 insensitive to MCC950/CRID3 inhibition.

Our results in *Nlrp3*^A350V^ macrophages showed that this mutation subtly altered the ability of MCC950/CRID3 to inhibit inflammasome activation with potent inhibition seen only in response to LPS but not following “signal 2” triggers such as ATP and nigericin (Fig 4). To determine how this translates to the in vivo disease setting, we analyzed circulating cytokine levels and body weight loss of *Nlrp3*^A350V/+^CreT^+^ mice following tamoxifen administration. Although there was a trend towards increased serum levels of IL-1β, the low measured concentrations did not reach statistical significance compared to tamoxifen-treated *Nlrp3*^A350V/+^CreT^−^ littermate mice (S1D Fig and S7 Data). However, serum concentrations of IL-18 and IL-6 were significantly elevated in tamoxifen-treated *Nlrp3*^A350V/+^CreT^+^ mice relative to *Nlrp3*^A350V/+^CreT^−^ littermates (S1E and S1F; S7 Data). Nevertheless, levels of the latter cytokines were on average 10- to 20-fold lower than seen in tamoxifen-treated *Nlrp3*^L351P/+^CreT^+^ mice, a finding that is consistent with the milder pathology and the lack of mortality associated with the *Nlrp3*^A350V/+^CreT^+^ CAPS model. Consistent with published findings [25], *Nlrp3*^A350V/+^CreT^+^ mice developed an inflammatory phenotype characterized by a steady weight loss of up to 20% within 27 days (Fig 6F and S5 Data). Notably, daily IP dosing of MCC950/CRID3 stabilized *Nlrp3*^A350V^-mediated body weight loss in *Nlrp3*^A350V/+^CreT^+^ mice relative to PBS-treated controls, although differences were small and MCC950/CRID3-treated mice failed to thrive and gain weight like the *Nlrp3*^A350V/+^CreT^−^ control group (Fig 6F and S5 Data). In agreement, MCC950/CRID3 had a mild or no effect on circulating levels of the systemic inflammatory markers IL-18 (Fig 6G and S5 Data) and IL-6 (Fig 6H and S5 Data), respectively. Together, these results establish that MCC950/CRID3 has a weak but measurable effect on Nlrp3^A350V^-induced inflammasomopathy in adult mice.

### MCC950/CRID3 inhibition of LPS-induced cytokines in CAPS mutant mice

To complement the chronic CAPS disease models described above, we next evaluated the potency of MCC950/CRID3 in inhibiting acute Nlrp3-dependent inflammasome responses by subjecting wild-type and CAPS mutant mice to LPS-induced endotoxemia and probing the effect of MCC950/CRID3 on well-documented Nlrp3-dependent readouts such as LPS-induced elevation of serum levels of IL-1β and IL-18 [26].

**Fig 7:**
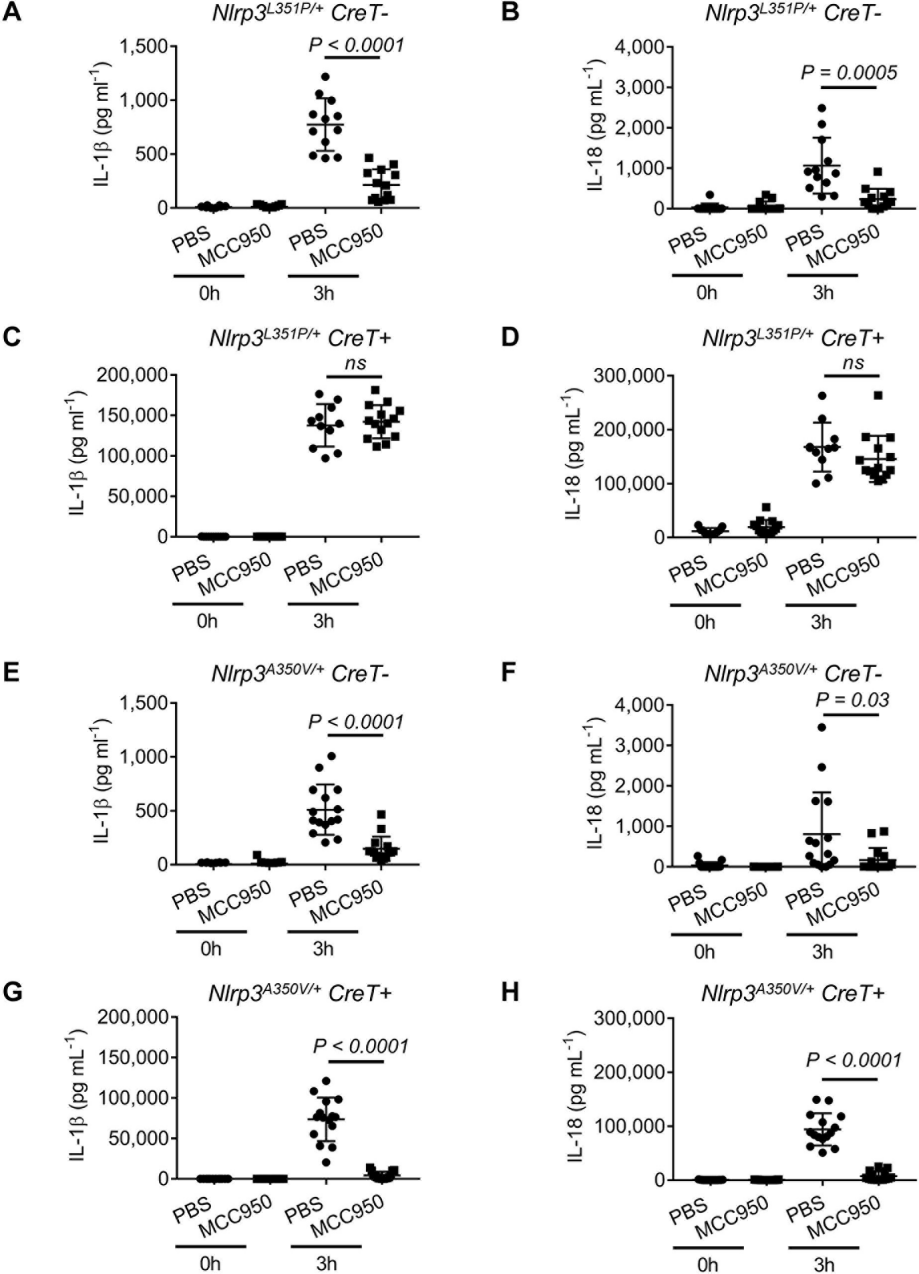
MCC950/CRID3 inhibition of LPS-induced cytokines in CAPS mutant mice. (A–B) At day 3 post-tamoxifen, *Nlrp3*^*L351P/+*^*CreT−* mice were pretreated with PBS (*n* = 12) or MCC950/CRID3 (*n* = 13) and subsequently challenged with LPS for 3 h. Serum levels of IL-1β (A) and IL-18 (B) were analyzed before and after LPS administration. (C–D) At day 3 post-tamoxifen, *Nlrp3*^*L351P/+*^*CreT+* mice were pretreated with PBS (*n* = 11) or MCC950/CRID3 (*n* = 14) and subsequently challenged with LPS for 3 h. Serum levels of IL-1β (C) and IL-18 (D) were analyzed before and after LPS administration. (E–F) At day 3 post-tamoxifen, *Nlrp3*^*A350V/+*^*CreT−* mice were pretreated with PBS (*n* = 15) or MCC950/CRID3 (*n* = 15) and subsequently challenged with LPS for 3 h. Serum levels of IL-1β (E) and IL-18 (F) were analyzed before and after LPS administration. (G–H) At day 3 post-tamoxifen, *Nlrp3*^*A350V/+*^*CreT+* mice were pretreated with PBS (*n* = 15) or MCC950/CRID3 (*n* = 15) and subsequently injected with LPS for 3 h. Serum levels of IL-1β (G) and IL-18 (H) were analyzed before and after LPS challenge. The numerical values underlying Fig 7A–7H can be found in S6 Data. Graphs show mean ± SD and represent two independent experiments. CAPS, cryopyrin-associated periodic syndrome; Cre-ERT2, Cre recombinase-estrogen receptor fusion protein; CreT, Cre-ERT2 fusion gene; CRID, Cytokine Release Inhibitory Drug; IL, interleukin; LPS, lipopolysaccharides; LRR, leucine-rich repeat; NBD, nucleotide-binding domain; NLR, NBD- and LRR-containing; NLRP3, NLR family, pyrin-domain–containing 3; ns, not significant.

As expected, *Nlrp3*^L351P/+^CreT^−^ control mice presented with increased serum levels of IL-1β and IL-18 3 h after LPS challenge (Fig 7A and 7B and S6 Data). MCC950/CRID3 substantially curbed circulating IL-1β and IL-18 levels in this control group (Fig 7A and 7B), consistent with reported findings in LPS-challenged C57BL/6 mice [15]. Contrastingly, serum levels of IL-1β and IL-18 in LPS-dosed *Nlrp3*^L351P/+^CreT^+^ mice were not significantly impacted by MCC950/CRID3 relative to PBS-treated *Nlrp3*^L351P/+^CreT^+^ littermates (Fig 7C and 7D and S6 Data).

LPS challenge increased serum concentrations of IL-1β and IL-18 in *Nlrp3*^A350V/+^CreT^−^ control mice, which were inhibited by MCC950/CRID3 (Fig 7E and 7F and S6 Data) similarly to its effect on LPS-treated *Nlrp3*^L351P/+^CreT^−^ control mice (Fig 7A and 7B and S6 Data). This is not unexpected because both genotypes express solely wild-type Nlrp3 in the absence of Cre-recombinase–mediated excision of the neomycin resistance cassette that disrupts expression of the respective CAPS-associated Nlrp3 mutants. However, in marked contrast to Nlrp3^L351P^-expressing CreT^+^ mice (Fig 7C and 7D and S6 Data), MCC950/CRID3 markedly curbed circulating concentrations of IL-1β and IL-18 in Nlrp3^A350V^-expressing CreT^+^ mice (Fig 7G and 7H). These results confirm that MCC950/CRID3 potently inhibits inflammasome activation by wild-type Nlrp3 and the MWS-associated Nlrp3^A350V^ mutant but not the FCAS-associated Nlrp3^L351P^ mutant.

## Discussion

Activation of the NLRP3 inflammasome has been observed in many diseases, and its central role in driving pathological inflammation renders it an attractive target for therapeutic intervention [10]. Gain-of-function mutations in NLRP3 cause hereditary periodic fever syndromes that are collectively referred to as CAPS, and these patients are currently treated with biologics that target secreted IL-1 [12]. As reported [27], chronic IL-1 blockade increases the risk for fatal infections and sepsis, suggesting that selective targeting of the NLRP3 inflammasome may potentially be a safer and more efficacious therapeutic strategy because it would block production of the central inflammatory mechanisms that contribute to inflammatory pathology while at the same time keeping nontargeted inflammasomes available to produce IL-1β to cope with infections.

Early studies with the sulfonylurea compound glyburide provided proof of concept that small molecules may selectively inhibit the NLRP3 inflammasome pathway without interfering with other inflammasomes [13]. Subsequently, several additional compounds have been reported to specifically inhibit the NLRP3 inflammasome pathway, but the majority of these agents have weak activity against the NLRP3 inflammasome pathway (μM IC_50_ concentrations) and may target nuclear factor κB (NF-κB) signaling and other immune pathways (reviewed in [10]). MCC950/CRID3 is structurally related to glyburide [14,15] and is considered the most potent and selective inhibitor of NLRP3 inflammasome signaling reported to date. There is substantial interest in developing this chemical scaffold for the treatment of CAPS and other diseases. However, these efforts are constrained by the lack of insight into the molecular target and mechanism by which MCC950/CRID3 and related sulfonylurea molecules inhibit activation of the NLRP3 inflammasome pathway.

Making use of PAL and iBody technology as complementary chemical biology approaches, we here identified NLRP3 as the physical target of MCC950/CRID3. We further mapped the binding pocket to the central NACHT domain of NLRP3 and showed that PAL required an intact ATP/dATP binding pocket. This suggests that binding may occur at the nucleotide-binding/hydrolysis pocket of NLRP3. This hypothesis is consistent with and extends results from two recent reports that suggest the adjacent Walker B motif as the putative binding site of MCC950/CRID3 [28,29]. However, it cannot be excluded that mutations in the Walker A/B motifs may cause long-distance conformational changes that distort a more remote MCC950/CRID3 binding pocket elsewhere in the NACHT domain. Notable in this regard is our observation that a randomly selected panel of 6 CAPS-associated gain-of-function mutations in human NLRP3 all failed to be labeled by our PAL probe, further supporting the notion that the sulfonylurea CRID binding pocket is highly sensitive for conformational changes in the protein that are imposed by mutations. Thus, further insight into the MCC950/CRID3 binding pocket and the molecular mechanism by which MCC950/CRID3 inhibits NLRP3 activation will likely require high-resolution structural analysis.

Considering the potential implications of these findings for treating CAPS with MCC950/CRID3-based therapies, we evaluated the functional impact of MCC950/CRID3 in two reported CAPS models [23]. When knocked into the murine Nlrp3 sequence, the FCAS-associated Nlrp3^L351P^ mutant (corresponding to human L353P) also failed to bind to PAL-CRID3. Consistent with these results, our analysis of both ex vivo-stimulated mutant macrophages and in vivo CAPS and endotoxemia models unequivocally established that MCC950/CRID3 potently inhibits inflammasome activation by wild-type Nlrp3 but not the FCAS-associated Nlrp3^L351P^ mutant. Surprisingly, however, we found that labeling by PAL-CRID3 to the MWS-linked Nlrp3^A350V^ mutant (corresponding to A352V in human NLRP3) was not significantly impacted by the mutation. MCC950/CRID3 partially inhibited LPS-induced IL-1β and IL-18 secretion from macrophages that homozygously or heterozygously expressed the Nlrp3^A350V^ mutant, but inhibition of the Nlrp3^A350V^ inflammasome was completely lost in response to “signal 2” agents ATP and nigericin. Consequently, MCC950/CRID3 significantly lowered IL-1β and IL-18 levels in serum of LPS-challenged *Nlrp3*^A350V^ knock-in mice, whereas it provided only limited protection against chronic CAPS mutation-driven body weight loss. The Cre-ERT2 recombinase expression system used with our CAPS disease models allows for controlled ubiquitous tissue expression of the mutant Nlrp3 knock-in allele upon tamoxifen treatment in adult animals. Expression of the *Nlrp3*^A350V^ allele does not induce lethality in adult mice under these conditions. However, another study [15] that relied on lysosome M-Cre–driven expression of the *Nlrp3*^A350V^ allele in cells of the myeloid lineage observed that MCC950/CRID3 rescued neonatal lethality, consistent with our observation that the Nlrp3^A350V^ mutant retained sensitivity to MCC950/CRID3 inhibition. This report also suggested MCC950/CRID3 inhibits LPS-induced IL-1β processing in peripheral blood mononuclear cells (PBMCs) from MWS patients. Another study showed that unlike samples from healthy controls, LPS- and LPS + ATP-induced IL-1β secretion from whole-blood samples of genetically defined CAPS patients resisted MCC950/CRID3 inhibition [30], which is consistent with our results suggesting that MCC950/CRID3-based therapies may effectively treat inflammation driven by wild-type NLRP3 but may be markedly less effective in CAPS patients. To conclude, by identifying the molecular target of MCC950/CRID3 in the NLRP3 inflammasome pathway and by evaluating its ability to inhibit CAPS mutant variants, the findings presented here provide a mechanistic framework for advancing therapeutic development of this chemical scaffold and for understanding its therapeutic potential in patients.

## Material and methods

### Ethics statement

All animal experiments were conducted with permission of the ethical committee on laboratory animal welfare of Ghent University. All procedures were performed in accordance with the guidelines and regulations associated with protocol number EC2017-090.

### Synthesis of PAL-CRID3

Detailed experimental procedures for the synthesis of PAL-CRID3 are provided in the Supplementary Data section (S1 Text).

### Synthesis of iBody conjugates

Experimental procedures for the preparation of the iBody conjugates have been described by [18], and details for the production of MCC950/CRID3 iBody conjugates are provided in the Supplementary Data section.

### iMac and BMDM culture

Bone marrow cells from C57BL/6 mice were immortalized as described previously [31]. Primary bone marrow or iMac progenitor cells were differentiated in DMEM or IMDM supplemented with 10% endotoxin-free heat-inactivated fetal bovine serum, 20%–30% L929-conditioned medium, 100 U ml^−1^ penicillin, and 100 mg ml^−1^ streptomycin for 5–6 days at 37°C in a humidified atmosphere containing 5% CO_2_. 6 days later, cells were collected and seeded at a density of 8.5 × 10^5^ cells per well in 12-well plates in IMDM containing 10% heat-inactivated FBS and 1% nonessential amino acids in the presence of antibiotics. The next day, BMDMs were either left untreated or treated with 1 μM MCC950/CRID3 (S7809; Selleckchem, Houston, TX, USA) and then stimulated with 0.5 μg ml^−1^ ultrapure LPS from *Salmonella minnesota* (tlrl-smlps; Invivogen, San Diego, CA, USA) for 3 h followed by 5 mM ATP (10519987001; Roche, Basel, Switzerland) or 20 μM nigericin (N-7143; Sigma-Aldrich, St. Louis, MO, USA) for 45 min. For the dose–response analysis of PAL-CRID3 and MCC950/CRID3, adherent BMDMs or differentiated iMac were seeded at 1 × 10^5^ cells per well in 96-well plates and cultured overnight. The following day, medium was removed and replaced with OPTI-MEM I (Thermo Fisher Scientific, Waltham, MA, USA) containing 1 μg mL^−1^ Pam3CSK4 (Invivogen). Postpriming (5–6 h later), cells were incubated with DMSO (1:1,000), MCC950/CRID3 (0.001–100 μM), or PAL-CRID3 (0.001–100 μM) for 30 min and then stimulated with 5 μg mL^−1^ nigericin (Invivogen) for 30 min.

### Transfections

HEK293T cells were maintained in DMEM media supplemented with 10% fetal bovine serum. HEK293T cells were transfected in 12-well plates with Lipofectamine 2000 (Thermo Fisher Scientific) according to manufacturer’s instructions. All constructs, including NLRP3 cDNAs were synthesized and subcloned into pCDNA3 (+) Zeo (Thermo Fisher Scientific).

### Photolabeling

Transfected HEK293T were incubated with DMSO or photo probe PAL-CRID3 (1 μM unless stated otherwise) for 30 min at 37°C in 500 μL OPTI-MEM. For competition experiments with free MCC950/CRID3, 10 μM MCC950/CRID3 was added 30 min prior. Photolabeling and click chemistry experiments were performed as described with slight modification [32]. Briefly, cells were washed once with ice-cold PBS and UV irradiated at 365 nm (100 W) on ice for 10 min. Cells were washed once with ice-cold PBS and frozen at −80°C. Lysis buffer was added (40 mM HEPES, 140 mM NaCl, 0.1% Triton-X-100, Roche EDTA-free complete protease inhibitor), and cells were detached with a cell scraper. Crude lysates were clarified by centrifugation (30 min at 14,000 rpm, 4°C). For click chemistry, lysates (20 μL) were incubated with 5 μL freshly mixed click cocktail composed of 1.7 mM TBTA in 1:4 DMSO/*t-*BuOH (1.5 μL), 5 mM TAMRA-N_3_ (0.3 μL), 50 mM TCEP (0.5 μL, freshly prepared), and 10% SDS (2.7 μL). Then, 50 mM CuSO_4_ (0.5 μL), was added, and reactions were incubated for 1 hr at RT with gentle mixing. Reactions were quenched with 4× LDS buffer and separated by SDS-PAGE. Gels were scanned for TAMRA fluorescence on a Typhoon Trio scanner (GE Life Sciences, Chicago, IL, USA).

### Mice

*Nlrp3*^*−/−*^ [33] and *NLRC4*^*3xFlag*^ [21] were described. The CAPS models *Nlrp3*^*A350VneoR*^ and *Nlrp3*^*L351PneoR*^ [23] and *R26-Cre*^*ERT2*^ mice [24] (B6.129-*Gt(ROSA)26Sor*^*tm1(cre/ERT2)Tyj*^/J, Jax stock number: 008463; here abbreviated as CreT) were originally obtained from The Jackson Laboratory (Bar Harbor, ME, USA), and colonies were further maintained at the animal facilities of Ghent University. Mice were housed in individually ventilated cages and kept under pathogen-free conditions at animal facilities of Ghent University. *Nlrp3*^*A350VneoR*^ and *Nlrp3*^*L351PneoR*^, which are homozygous for the mutated Nlrp3 gene, were bred to the tamoxifen-inducible Cre line *R26-Cre*^*ERT2*^ mice to generate *Nlrp3*^*A350neoR/+*^*R26-Cre*^*ERT2*^*Tg+* (herein referred to as *Nlrp3*^*A350V/+*^*CreT*^*+*^) and *Nlrp3*^*L351PneoR/+*^*R26-Cre*^*ERT2*^*Tg*^*+*^ mice (here referred to as *Nlrp3*^*L351P/+*^*CreT*^+^), in which expression of mutant Nlrp3 is induced through administration of tamoxifen. 4- to 5-week–old mice received at two consecutive days tamoxifen (T5648, Sigma-Aldrich, dissolved in 1:9 ethanol/corn oil (C-8267, Sigma-Aldrich) at 50 mg ml^−1^) through oral gavage at a dose of 5 mg tamoxifen per mouse per day. On day 3, tamoxifen was administered through diet (Teklad Global 16% Rodent Diet with 400 ppm tamoxifen per kg; Harlan, Horst, The Netherlands).

### In vivo LPS challenge

6- to 12-week–old mice were intraperitoneally injected with PBS or 50 mg kg^−1^ MCC950/CRID3, 30 minutes before being challenged with 40 mg kg^−1^ LPS (*Escherichia coli*, serotype 0111:B4, L-2630; Sigma-Aldrich). Mice were euthanized 3 h after LPS challenge for blood collection. At the 0 h time point before LPS challenge, blood was collected by retro-orbital bleeding.

### iBody immunoprecipitation

12 × 10^6^ BMDMs were harvested, and cells were washed in PBS and lysed by three cycles of freeze/thawing in PBS with 0.09% NP40 supplemented with complete protease inhibitor cocktail (4693159001; Roche Applied Science). Cell lysates were clarified by centrifugation at 14,000 rpm for 20 minutes, and supernatants were subsequently incubated with 1 μM of the indicated iBody at RT for 10 minutes. Then prewashed streptavidin conjugated beads were added, followed by overnight incubation at 4°C. The next day, beads were washed 3 times in PBS with 0.09% NP40 buffer, and biotinylated proteins were eluted in Laemmli buffer and analyzed by western blot.

### Lentiviral Cre transduction

To induce lentivirus production, the lentiviral GFP.Cre empty vector (20781; Addgene, Watertown, MA, USA), together with second-generation packaging vector psPAX2 (12260; Addgene) and VSV-G–expressing envelope plasmid pCMV-VSV-G (8454; Addgene), was transfected into HEK293T cells using jetPRIME transfection reagent (114–15; PolyPlus-transfection, Illkirch, France). Lentiviral-particle–containing medium was collected 48 h after transfection, filtered using a 0.45 μm filter, and incubated with harvested bone marrow. 6 days later, differentiated macrophages were collected, washed, and seeded into 12-well cell culture plates prior to stimulation.

### Cytokine analysis

Cytokine levels in culture medium and serum were determined by magnetic-bead–based multiplex assay using Luminex technology (Bio-Rad, Hercules, CA, USA), IL-1β ELISA (R&D Systems, Minneapolis, MN, USA) and mouse IL-1β tissue culture kit (Meso Scale Discovery, Rockville, MD, USA) according to the manufacturers’ instructions.

### Western blotting

Cell lysates were prepared using lysis buffer containing 20 mM Tris HCl (pH 7.4), 200 mM NaCl, and 1% NP-40. Samples for detection of Casp1 and IL-1β processing were prepared by combining cell lysates with culture supernatants. Samples were denatured in Laemmli buffer and boiled at 95°C for 10 min. SDS-PAGE–separated proteins were transferred to PVDF membranes and immunoblotted with primary antibodies against Casp1 (AG-20B-0042-C100; Adipogen, Liestal, Switzerland), IL-1β (GTX74034; Genetex, Irvine, CA, USA), Flag-tag (Flag M2-Peroxidase; Sigma-Aldrich) and actin (AC15; Novus Biologicals, Centennial, CO, USA). Horseradish-peroxidase–conjugated goat anti-mouse (115-035-146; Jackson Immunoresearch Laboratories, West Grove, PA, USA) or anti-rabbit secondary antibody (111-035-144; Jackson Immunoresearch Laboratories) was used to detect proteins by enhanced chemiluminescence (Thermo Fisher Scientific).

### Statistical analysis

GraphPad Prism 5.0 software was used for data analysis. For survival studies, data were compared by log-rank (Mantel–Cox) test. Two-way ANOVA tests were used to assess body weight differences between groups. Unpaired two-tailed Student *t* test was applied to compare cytokine serum levels. Data are shown as mean with standard deviation. *P* < 0.05 was considered to indicate statistical significance.

## Supporting information

S1 Fig Tamoxifen-inducible mouse models of CAPS. (A–C) Serum levels of IL-1β (A), IL-18 (B), and IL-6 (C) in *Nlrp3*^*L351P/+*^*CreT+* and *CreT−* littermates before and after tamoxifen treatment at day 3. (D–E) Serum levels of IL-1β (D), IL-18 (E), and IL-6 (F) in *Nlrp3*^*A350V/+*^*CreT+* and *CreT−* littermates before and after tamoxifen treatment at day 3. CAPS, cryopyrin-associated periodic syndrome; Cre-ERT2, Cre recombinase-estrogen receptorfusion protein; CreT, Cre-ERT2 fusion gene; IL, interleukin; LRR, leucine-rich repeat; NBD, nucleotide-binding domain; NLR, NBD- and LRR-containing; NLRP3, NLR family, pyrin-domain–containing 3
(JPG)

S2 Fig Synthesis of compound 2. (A–B) Synthesis (A) and UPLC/MS analysis (B) of mixture of isomer compound 2 (PSI213) (total ion chromatogram, 210 nm chromatogram, mass [655]^++^ chromatogram). UPLC/MS, ultra-performance liquid chromatography/mass spectrometry.
(JPG)

S3 Fig GPC chromatogram of polymer precursor (blue), polymer conjugate U-121 (orange), and U-126 (gray). GPC, gel permeation chromatography.
(JPG)

S1 Text Supplementary materials and methods. (DOCX)

S1 Data Numerical data underlying Fig 1B and 1C. (XLSX)

S2 Data Numerical data underlying Fig 3A, 3B, 3D, 3E, 3G and 3H. (XLSX)

S3 Data Numerical data underlying Fig 4A, 4B, 4D, 4E, 4G and 4H. (XLSX)

S4 Data Numerical data underlying Fig 5A, 5B, 5C, 5D, 5E and 5F. (XLSX)

S5 Data Numerical data underlying Fig 6A, 6B, 6C, 6D, 6E, 6F, 6G and 6H. (XLSX)

S6 Data Numerical data underlying Fig 7A, 7B, 7C, 7D, 7E, 7F, 7G and 7H. (XLSX)

S7 Data Numerical data underlying S1A, S1B, S1C, S1D, S1E and S1F Fig. (XLSX)

## Abbreviations

ASC: apoptosis-associated speck-like protein
BMDM: bone-marrow–derived macrophage
CAPS: cryopyrin-associated periodic syndrome
Casp1: caspase-1
CPPD: calcium pyrophosphate dihydrate
CINCA: Chronic Infantile Neurological, Cutaneous, and Articular Syndrome
Cre-ERT2: Cre recombinase-estrogen receptor fusion protein
CreT: Cre-ERT2 fusion gene
CRID: Cytokine Release Inhibitory Drug
DAMP: danger-associated molecular pattern
ER-Hoxb8: estrogen-regulated homeobox protein Hox-B8
FCAS: Familial Cold Autoinflammatory Syndrome
GSDMD: gasdermin D
GSTO1: Glutathione S-transferase omega-1
HD: helical domain
HEK: human embryonic kidney
HPMA: *N*-(2-hydroxypropyl)methacrylamide
IC_50_: half maximal inhibitory concentration
IL: interleukin
iMac: ER-Hoxb8–immortalized macrophages
IP: intraperitoneal
LPS: lipopolysaccharides
LRR: leucine-rich repeat
MEFV: Mediterranean fever
MSU: monosodium urate
MWS: Muckle–Wells syndrome
NACHT: NAIP, CIITA, HET-E, and TP1
NAFLD: nonalcoholic fatty liver disease
NASH: nonalcoholic steatohepatitis
NBD: nucleotide-binding domain
NEK7: never-in-mitosis-gene-a–related kinase 7
NF-κB: nuclear factor κB
NLR: NBD- and LRR-containing
Nlrc4: NLR family, caspase-recruitment-domain–containing 4
NLRP3: NLR family, pyrin-domain–containing 3
NOD: nucleotide-binding oligomerization domain
NOMID: Neonatal Onset Multisystem Inflammatory Disease
ns: not significant
PAL: photoaffinity labeling
PAMP: pathogen-associated molecular pattern
Pam3CSK4: N-palmitoyl-S-dipalmitoylglyceryl Cys-Ser-(Lys)4
PBMC: peripheral blood mononuclear cell
PP1Cb: protein phosphatase 1 catalytic subunit β isoform
PYD: Pyrin domain
TAMRA: 5-carboxytetramethylrhodamine
WHD: winged helix domain
