# Correction: MCC950/CRID3 potently targets the NACHT domain of wild-type NLRP3 but not disease-associated mutants for inflammasome inhibition

**DOI:** 10.1371/journal.pbio.3000529

**Published:** 2019-10-16

**Authors:** Lieselotte Vande Walle, Irma B. Stowe, Pavel Šácha, Bettina L. Lee, Dieter Demon, Amelie Fossoul, Filip Van Hauwermeiren, Pedro H. V. Saavedra, Petr Šimon, Vladimír Šubr, Libor Kostka, Craig E. Stivala, Victoria C. Pham, Steven T. Staben, Sayumi Yamazoe, Jan Konvalinka, Nobuhiko Kayagaki, Mohamed Lamkanfi

The tenth author's name is spelled incorrectly. The correct name is: Vladimír Šubr.
