# Supplementary figures and images for: MCC950/CRID3 potently targets the NACHT domain of wild-type NLRP3 but not disease-associated mutants for inflammasome inhibition
Source: PLoS Biol. 2019 Sep 16;17(9):e3000354. doi: 10.1371/journal.pbio.3000354 (PMC6762198; doi:10.1371/journal.pbio.3000354)

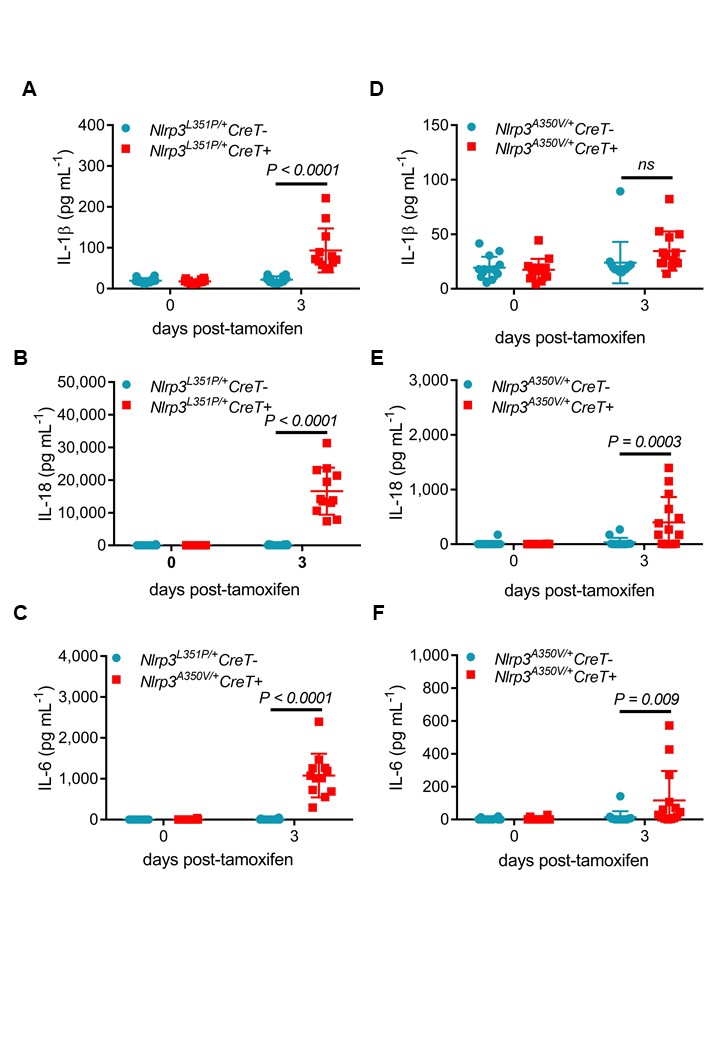

Supplement: S1 Fig — (A–C) Serum levels of IL-1β (A), IL-18 (B), and IL-6 (C) in Nlrp3L351P/+CreT+ and CreT− littermates before and after tamoxifen treatment at day 3. (D–E) Serum levels of IL-1β (D), IL-18 (E), and IL-6 (F) in Nlrp3A350V/+CreT+ and CreT− littermates before and after tamoxifen treatment at day 3. CAPS, cryopyrin-associated periodic syndrome; Cre-ERT2, Cre recombinase-estrogen receptorfusion protein; CreT, Cre-ERT2 fusion gene; IL, interleukin; LRR, leucine-rich repeat; NBD, nucleotide-binding domain; NLR, NBD- and LRR-containing; NLRP3, NLR family, pyrin-domain–containing 3 (JPG) [file pbio.3000354.s001.JPG]

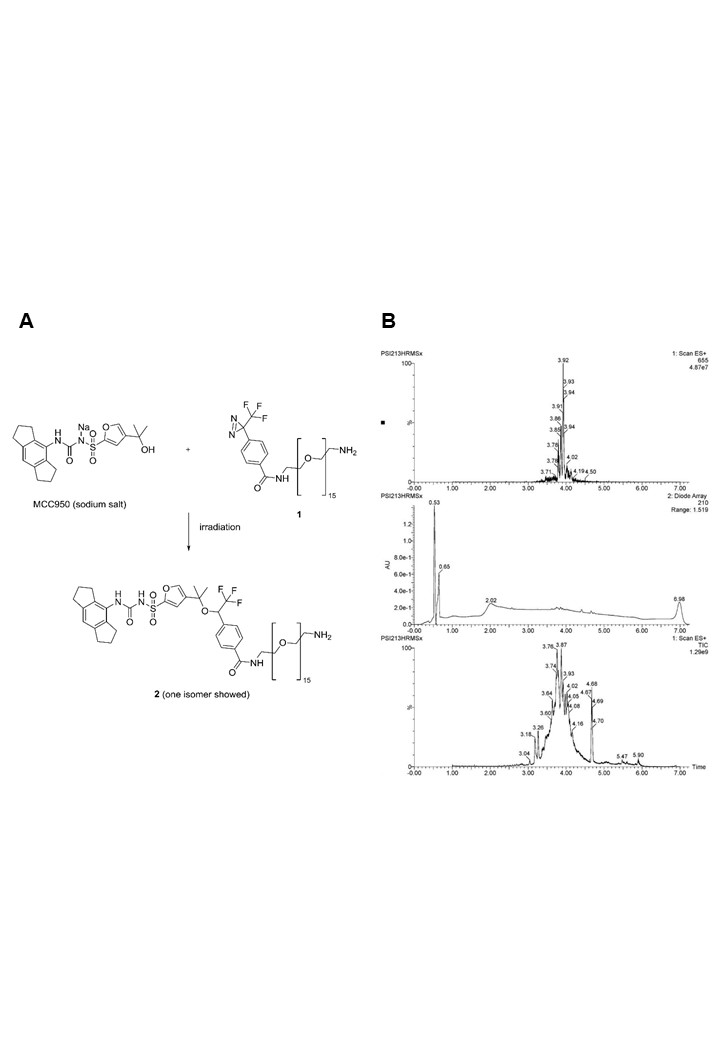

Supplement: S2 Fig — (A–B) Synthesis (A) and UPLC/MS analysis (B) of mixture of isomer compound 2 (PSI213) (total ion chromatogram, 210 nm chromatogram, mass [655]++ chromatogram). UPLC/MS, ultra-performance liquid chromatography/mass spectrometry. (JPG) [file pbio.3000354.s002.JPG]

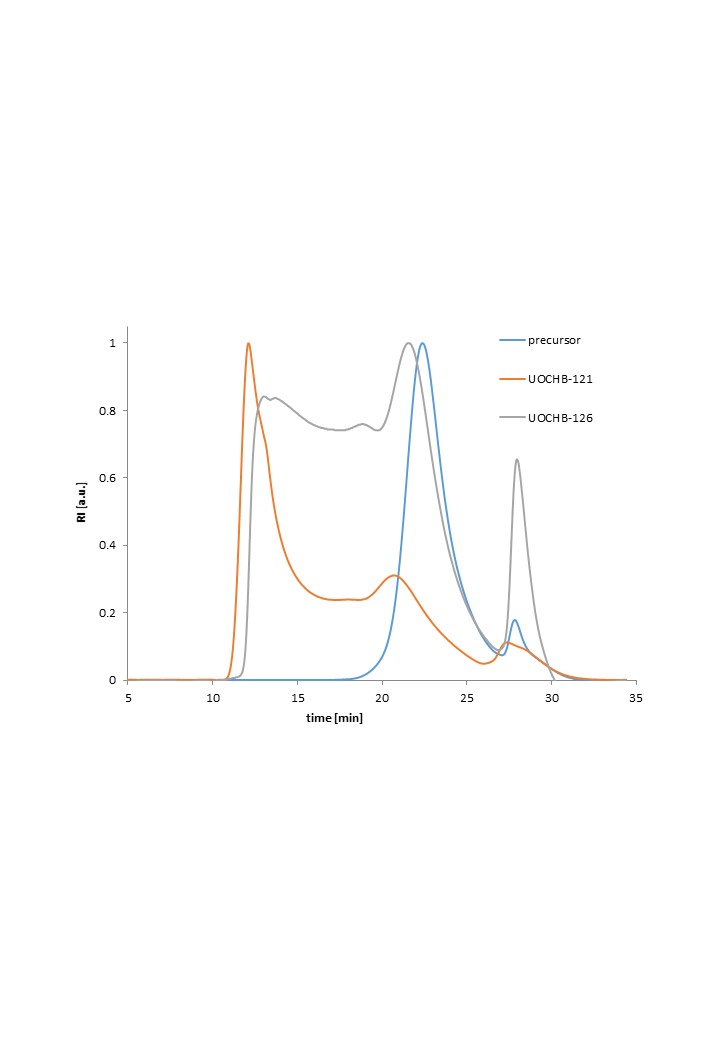

Supplement: S3 Fig — GPC, gel permeation chromatography. (JPG) [file pbio.3000354.s003.JPG]
