# Supplementary material for: MCC950/CRID3 potently targets the NACHT domain of wild-type NLRP3 but not disease-associated mutants for inflammasome inhibition
Source: PLoS Biol. 2019 Sep 16;17(9):e3000354. doi: 10.1371/journal.pbio.3000354 (PMC6762198; doi:10.1371/journal.pbio.3000354)
Supplement: S1 Text — (DOCX) [file pbio.3000354.s004.docx]

**Supplementary Materials & Methods**

Synthesis of PAL-CRID3:

All reactions were carried out under a nitrogen atmosphere. All commercial reagents and anhydrous solvents were used without additional purification. Nuclear magnetic resonance (NMR) spectra were acquired on a Bruker BioSpin GmbG operating at 400 and 100 MHz for ^1^H and ^13^C, respectively and are referenced internally according to residual solvent signals. NMR data were processed using MNova software and recorded as follows: ^1^H-NMR - chemical shift (δ, ppm), multiplicity (s, singlet; d, doublet; t, triplet; q, quartet; m, multiplet), coupling constant (Hz), and integration; ^13^C-NMR – chemical shift (δ, ppm). High-resolution mass spectra (HRMS) were recorded on a Thermo Scientific Orbitrap Q Exact mass spectrometer. Thin-layer chromatography was performed on EMD TLC Silica gel 60 F254 plates and visualized with UV light. Reactions were monitored by a Shimadzu LCMS/UV system with LC-30AD solvent pump, 2020 MS, Sil-30AC autosampler, SPD-M30A UV detector, CTO-20A column oven, using a 2-98% acetonitrile/0.1% formic acid (or 0.001% ammonia) gradient over 2.5 minutes. Flash column chromatography purifications were done on a Teledyne Isco Combiflash Rf utilizing Silicycle HP columns using a mobile phase composed of either heptane/isopropyl acetate or dichloromethane/methanol.

Preparation of tert-Butyl (3-(3-sulfamoylphenoxy)propyl)carbamate:

^^

Potassium carbonate (1.00 g, 7.5 mmol) was added to a solution of 3-hydroxybenzenesulfonamide (1.0 g, 5.8 mmol) and 3-(Boc-amino)propyl bromide (1.40 g, 5.8 mmol) in N,N-dimethylformamide (14 mL). The reaction was sealed with a yellow cap and heated at 55^o^C for 16h. After cooling to room temperature, the reaction was diluted with water and isopropyl acetate. The aqueous layer was extracted with isopropyl acetate (3 x 30 mL). The combined organic layers were dried with sodium sulfate, concentrated and the crude residue was purified by flash column chromatography (silica, 100% isopropyl acetate) to give tert-butyl (3-(3-sulfamoylphenoxy)propyl)carbamate (1.34 g, 4.06 mmol, 70% Yield).

^1^H NMR (400 MHz, DMSO-*d*_6_) δ 7.47 (t, *J* = 7.9 Hz, 1H), 7.42 – 7.33 (m, 2H), 7.32 (s, 2H), 7.14 (ddd, *J* = 8.2, 2.5, 1.0 Hz, 1H), 6.90 (t, *J* = 5.6 Hz, 1H), 4.03 (t, *J* = 6.3 Hz, 2H), 3.09 (q, *J* = 6.6 Hz, 2H), 1.85 (p, *J* = 6.5 Hz, 2H), 1.37 (s, 9H).

^13^C NMR (101 MHz, DMSO) δ 159.07, 156.12, 145.84, 130.56, 118.48, 118.03, 111.84, 78.00, 66.17, 37.30, 29.54, 28.72.

HRMS (ESI) calcd for C_14_H_22_N_2_O_5_S [M-H]^+^: 329.1177, found: 329.1179.

Preparation of 4-(4-(Prop-2-yn-1-yloxy)benzoyl)-*N*-(3-(3-sulfamoylphenoxy)propyl)benzamide:

Trifluoroacetic acid (3.40 mL, 45.4 mmol) was added to a solution of tert-butyl (3-(3-sulfamoylphenoxy)propyl)carbamate (0.750 g, 2.27 mmol) in dichloromethane (45 mL) at room temperature. After 1 h, the reaction was concentrated under reduced pressure. Toluene (50 mL) was added and the reaction was again concentrated under reduced pressure. The crude residue was then submitted to the next step without further purification.

4-Dimethylaminopyridine (6.8 mg, 0.054 mmol) was added to a solution of 3-(3-aminopropoxy)benzenesulfonamide (92 mg, 0.401 mmol), 4-(4-prop-2-ynoxybenzoyl)benzoic acid (75 mg, 0.268 mmol), 1-(3-dimethylaminopropyl)-3-ethylcarbodiimide hydrochloride (62.2 mg, 0.321 mmol) and N,N-diisopropylethylamine (0.140 mL, 0.803 mmol) in N,N-dimethylformamide (2.7 mL) at room temperature. After 3 days, the reaction was diluted with water and isopropyl acetate and the aqueous layer was acidified to pH 1 using 1M HCl. The aqueous layer was extracted with isopropyl acetate (3 x 50 mL). The combined organic layers were dried with sodium sulfate, concentrated and the crude residue was purified by flash column chromatography (silica, 100% isopropyl acetate) to give 4-(4-(prop-2-yn-1-yloxy)benzoyl)-*N*-(3-(3-sulfamoylphenoxy)propyl)benzamide (46 mg, 0.093 mmol, 35% Yield).

^1^H NMR (400 MHz, DMSO-*d*_6_) δ 8.76 (t, *J* = 5.6 Hz, 1H), 8.03 – 7.96 (m, 2H), 7.82 – 7.73 (m, 4H), 7.48 (t, *J* = 7.9 Hz, 1H), 7.43 – 7.35 (m, 2H), 7.34 (s, 2H), 7.21 – 7.12 (m, 3H), 4.94 (d, *J* = 2.4 Hz, 2H), 4.12 (t, *J* = 6.1 Hz, 2H), 3.65 (t, *J* = 2.3 Hz, 1H), 3.49 (q, *J* = 6.4 Hz, 2H), 2.04 (p, *J* = 6.5 Hz, 2H).

^13^C NMR (101 MHz, DMSO) δ 194.49, 166.12, 161.52, 159.08, 145.86, 140.25, 137.95, 132.59, 130.60, 130.26, 129.62, 127.76, 118.51, 118.07, 115.29, 111.88, 79.27, 79.13, 66.29, 56.26, 36.83, 29.21.

HRMS (ESI) calcd for C_26_H_25_N_2_O_6_S [M+H]^+^: 493.1428, found: 493.1418.

Preparation of 4-Isocyanato-1,2,3,5,6,7-hexahydro-s-indacene:

Triphosgene (88 mg, 0.288 mmol) was added in one portion to a solution of 1,2,3,5,6,7-hexahydro-s-indacen-4-amine (0.150 g, 0.866 mmol) and triethylamine (0.130 mL, 0.91 mmol) in THF (2.90 mL) and the mixture was heated to reflux. After 2 h the reaction was cooled to room temperature and the volatiles were removed under reduced pressure. The crude residue was dissolved in pentane and filtered through a plug of silica gel (to remove the triethylammonium chloride). The filtrate was concentrated under reduced pressure and the crude residue was submitted to the next step without further purification.

Preparation of Sodium ((1,2,3,5,6,7-hexahydro-*s*-indacen-4-yl)carbamoyl)((3-(3-(4-(4-(prop-2-yn-1-yloxy)-benzoyl)benzamido)propoxy)phenyl)sulfonyl)amide:

Sodium hydroxide (10 wt% in water, 0.040 mL, 0.102 mmol) was added to a solution of 4-(4-(prop-2-yn-1-yloxy)benzoyl)-*N*-(3-(3-sulfamoylphenoxy)propyl)benzamide (50 mg, 0.102 mmol) in acetone and the reaction was heated at reflux for 20 min. The reaction was cooled to room temperature and the volatiles were removed under reduced pressure. The crude residue was then placed on a high vacuum for 1 h to remove residual water, then it was dissolved in acetone (1.0 mL) and the reaction was heated at reflux. A solution of 4-isocyanato-1,2,3,5,6,7-hexahydro-s-indacene (27 mg, 0.137 mmol) in acetone (0.4 mL total with rinses) was added dropwise to the refluxing solution over 5 min. After addition, the reaction was allowed to reflux for an additional 10 min. The reaction was cooled to room temperature and the solvent was removed via syringe. Acetone (2.0 mL) was added to the solid, the mixture was sonicated for 10 sec and the solvent was removed via syringe. This sequence was repeated twice using heptane. The solid was isolated and dried under vacuum to give sodium ((1,2,3,5,6,7-hexahydro-*s*-indacen-4-yl)carbamoyl)((3-(3-(4-(4-(prop-2-yn-1-yloxy)benzoyl)benzamido)propoxy)phenyl)sulfonyl)amide: (32 mg, 0.045 mmol, 44% Yield).

^1^H NMR (400 MHz, DMSO-*d*_6_) δ 8.78 (t, *J* = 5.5 Hz, 1H), 8.03 – 7.95 (m, 2H), 7.81 – 7.70 (m, 4H), 7.41 – 7.35 (m, 2H), 7.32 (dt, *J* = 7.7, 1.4 Hz, 1H), 7.25 (t, *J* = 7.9 Hz, 1H), 7.19 – 7.10 (m, 2H), 6.92 (ddd, *J* = 8.0, 2.6, 1.2 Hz, 1H), 6.73 (s, 1H), 4.93 (d, *J* = 2.4 Hz, 2H), 4.06 (t, *J* = 6.2 Hz, 2H), 3.64 (t, *J* = 2.4 Hz, 1H), 3.46 (q, *J* = 6.5 Hz, 2H), 2.71 (t, *J* = 7.4 Hz, 4H), 2.63 (t, *J* = 7.3 Hz, 4H), 2.02 (q, *J* = 6.4 Hz, 2H), 1.87 (p, *J* = 7.5 Hz, 4H).

^13^C NMR (101 MHz, DMSO) δ 194.50, 166.10, 161.51, 159.18, 158.42, 149.29, 142.59, 140.19, 137.94, 137.28, 133.01, 132.59, 130.27, 129.59, 129.18, 127.79, 118.76, 116.20, 116.14, 115.28, 112.55, 79.27, 79.13, 65.92, 56.27, 36.91, 33.05, 30.98, 29.33, 25.53.

HRMS (ESI) calcd for C_39_H_38_N_3_O_7_S [M+H]^+^: 692.2415, found: 692.2425.

Synthesis of iBodies:

The HPMA polymer conjugates, named iBody U-121 and U-126, were prepared by reaction of the polymer precursor poly(HPMA-co-Ma-ß-Ala-TT) with a combination of the affinity anchor *N*-(2-aminoethyl)biotinamide hydrobromide (biotin-NH2) and the targeting ligand. Polymer precursors poly(HPMA-co-Ma-ß-Ala-TT) were synthesized as described earlier [1]. In iBody U-121, the targeting ligand is compound 2, in which MCC950 is connected to a linker, compound 1, prepared as described [2]. In iBody U-126, the targeting ligand is the GCPII inhibitor modified with a diazirine linker, which is prepared as described [2].

Preparation of compound 2. MCC950 (sodium salt, 52 mg, 0.122 mmol) and compound 1 (62 mg, 0.066 mmol) were dissolved in methanol and evaporated to dryness *in vacuo*. The solid residue was irradiated using LED diode (365 nm, 1 W) until starting compound 1 was present (UPLC/MS analysis). The reaction mixture was dissolved in 80% methanol and subjected to Sephadex LH-20 gel chromatography. Fractions containing desired product (UPLC/MS analysis) were collected, evaporated to dryness, and purified using preparative HPLC. Fractions containing desired product (UPLC/MS analysis) were collected, evaporated to dryness, and used in reaction with HPMA polymer. Mixture of isomers compound 2 was isolated as white solid (5 mg). UPLC/MS (m/z) 655.33 [M+2H]^++^, Tr 3.56-4.19 min; HRMS (ESI): Calculated for C_61_H_96_F_3_N_4_O_21_S [M+H]^+^: 1309.6240, Found: 1309.6220 (Supplemental Figure S2).

Preparation of iBody U-121. Polymer precursor poly(HPMA-*co*-Ma-β-Ala-TT) (15 mg), compound 2 (PSI137g3) (3.0 mg) and biotin-NH_2_ (1.5 mg) were dissolved in 0.3 ml DMSO and *N*,*N*-diisopropylethylamine (DIPEA) (2.3 µl) was added. The reaction mixture was stirred at room temperature for 4.5 h and then 2 µl of 1-aminopropan-2-ol was added and the reaction was again stirred for 10 min. Solution of polymer conjugate poly(HPMA-*co*-Ma-β-Ala-compound 2-Ma-β-Ala-NH-biotin) was diluted with 0.7 ml of methanol and purified on chromatography column Sephadex LH-20 in methanol equipped with UV detector (220 nm). Polymer conjugate was dissolved in 1.5 ml of distilled water and lyophilized. Yield of U-121 (M_n_ = 303,000 g/mol, M_w_ = 982,000 g/mol, Ð = 3.24) was 13 mg, content of biotin was 3.3 wt% (Supplemental Figure S2).

Preparation of iBody U-126. Polymer precursor poly(HPMA-*co*-Ma-β-Ala-TT) (10 mg), GCPII inhibitor modified with a diazirine linker (3.0 mg), biotin-NH_2_ (1.1 mg) and ATTO488-NH_2_ (0.8 mg) were dissolved in 0.5 ml DMSO, followed by the addition of DIPEA (5.2 µl). Reaction was carried out for 4.5 h at room temperature and then 2 µl of 1-aminopropan-2-ol was added and the reaction was stirred for 10 min. Solution of polymer conjugate was diluted with 1.5 ml of methanol and purified on chromatography column Sephadex LH-20 in methanol. Methanol was evaporated and polymer conjugate was dissolved in 1.5 ml of distilled water and purified on chromatography column Sephadex G-25 and lyophilized. Yield of U-126 (M_n_ = 193,500 g/mol, M_w_ = 685,000 g/mol, Ð = 3.54) was 9 mg, content of biotin was 3.5 wt% (Supplemental Figure 3).

**References to supplementary data**

1. Šubr V, Ulbrich K. Synthesis and properties of new N-(2-hydroxypropyl) methacrylamide copolymers containing thiazolidine-2-thione reactive groups. Reactive and Functional Polymers. 2006;66(12):1525-38.

2. Simon P, Knedlik T, Blazkova K, Dvorakova P, Brezinova A, Kostka L, et al. Identification of Protein Targets of Bioactive Small Molecules Using Randomly Photomodified Probes. ACS Chem Biol. 2018;13(12):3333-42. doi: 10.1021/acschembio.8b00791. PubMed PMID: 30489064.
